# Light-controlled pyroptosis via redox-responsive microneedles enhances photodynamic–epigenetic immunotherapy in breast cancer

**DOI:** 10.1016/j.mtbio.2025.102158

**Published:** 2025-07-31

**Authors:** Hang Yu, Yiqing Chen, Jinjin Yin, Zhongwen Yuan, Senling Feng, Yanrong Duan, Pengke Yan, Shengyao Liu, Wenting Zhu

**Affiliations:** a Department of Pharmacy, Biomedicine Research Center, Guangdong Provincial Key Laboratory of Major Obstetric Diseases, Guangdong Provincial Clinical Research Center for Obstetrics and Gynecology, The Third Affiliated Hospital, Guangzhou Medical University, Guangzhou, 510150, China; b Department of Neurosurgery and Neurosurgical Disease Research Centre, The Second Affiliated Hospital of Guangzhou Medical University, Guangzhou, 510006, China; c Department of Spine Surgery, The Second Affiliated Hospital of Guangzhou Medical University, Guangzhou, 510260, China

**Keywords:** Soluble microneedle, Pyroptosis, Photodynamic therapy, Glutathione depletion, Breast cancer

## Abstract

Despite the potential of photodynamic therapy in breast cancer treatment, inadequate immunogenicity and inefficient pyroptosis induction remain critical limitations. To address this, we developed dissolvable microneedles (MNs) for localized co-delivery of decitabine (DEC) and glutathione (GSH)-responsive photosensitizer nanoparticles (HPPH-ss-NPs), aiming to potentiate immunogenic pyroptosis in breast cancer. The MNs enabled spatiotemporal control of DEC (dissolution-dependent release) and HPPH-ss-NPs (GSH-triggered activation), enhancing tumor drug levels with reduced systemic exposure. Mechanistically, DEC restored pyroptosis executioner gasdermin E (GSDME) expression, while HPPH-ss-NPs depleted intracellular GSH and generated caspase-3-activating ROS under light irradiation. The synergistic action triggered GSDME-dependent pyroptosis, releasing immunostimulatory DAMPs that increased mature dendritic cells and tumor-infiltrating CD8^+^ T cells. In orthotopic models, (DEC + HPPH-ss-NPs)@MNs suppressed primary tumor growth, while combining with anti-PD-1 synergistically inhibited tumor recurrence and lung metastasis, establishing durable systemic immunity. This MN-based platform establishes a localized photodynamic-epigenetic crosstalk to convert immunologically inert tumors into pyroptosis-driven immunogenic niches, providing a strategy for combinatorial breast cancer therapy.

## Graphical abstract

This study designs a dissolvable microneedle platform co-delivering decitabine and redox-responsive HPPH nanoparticles to enable spatiotemporally controlled photodynamic-epigenetic therapy. Laser-triggered pyroptosis and immunogenic cell death remodel immunosuppressive tumor niches, synergizing with PD-1 blockade to suppress primary/metastatic tumors and prevent recurrence.

**Figure:**
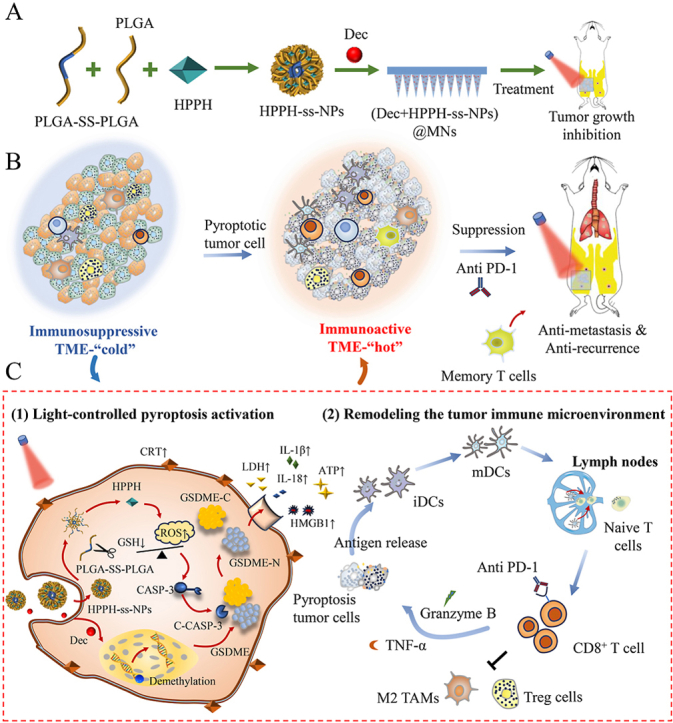

## 1 Introduction

Breast cancer continues to threaten women's health due to its high heterogeneity and aggressiveness. Although surgery combined with radiotherapy and chemotherapy can control primary lesions, patients still face high risks of recurrence and lung metastasis. Immunotherapy demonstrates clinical potential by activating adaptive immune responses. However, its efficacy is limited by the insufficient presence of tumor-specific antigens, the immunosuppressive tumor microenvironment, and inadequate tumor drug accumulation resulting from systemic administration [1,2]. The development of novel multifunctional synergistic strategies enabling precise delivery is urgently required.

Photodynamic therapy (PDT), a non-invasive modality utilizing photosensitizer-generated reactive oxygen species (ROS), faces three challenges, metabolic reprogramming-induced glutathione (GSH) overexpression that systematically neutralizes ROS, off-target phototoxicity from poor photosensitizer specificity, and reduced bioavailability due to hydrophobic aggregation [[3], [4], [5]]. The second-generation photosensitizer HPPH (3-(1′-hexyloxyethyl) pyropheophorbide-a) exhibits enhanced safety with reduced phototoxicity. Under 660 nm laser activation, it enables efficient ROS generation at minimal therapeutic doses for targeted tumor ablation while significantly reducing systemic side effects [6,7]. Furthermore, nanocarrier research enables targeted photosensitizer delivery while actively remodeling the tumor microenvironment (TME) through oxygen supplementation or GSH depletion, thereby potentiating PDT efficacy [8]. For instance, catalase-mimetic nanoparticles alleviate tumor hypoxia to enhance PDT efficacy [9], while disulfide bond-containing systems selectively deplete intratumoral GSH to amplify oxidative stress [10]. Furthermore, stimulus-responsive designs enable spatiotemporal control over therapeutic activation, minimizing systemic toxicity [11,12]. Liu et al. report smart nanosensitizers with tumor-targeting and redox-controlled disassembly for activatable sono-photodynamic immunotherapy of breast tumors and gliomas [13]. Research confirms that disulfide bonds enhance cellular uptake and cytoplasmic localization of polymer nanoparticles through interactions with surface thiols [14].To enhance the photodynamic efficacy of HPPH, we propose fabricating nanoparticles using high-performance polymer PLGA and its redox-sensitive derivative PLGA-SS-PLGA. The disulfide bridges in PLGA-SS-PLGA are designed to achieve tumor-targeted drug release under GSH-rich conditions while concurrently depleting GSH to amplify ROS effects.

PDT triggers immunogenic cell death (ICD) in tumors, releasing damage-associated molecular patterns (DAMPs) like CRT, ATP, and HMGB1 to activate dendritic cells (DCs) and prime CTL-mediated antitumor immunity, a process termed photodynamic immunotherapy (PDIT) [15,16]. However, residual tumors persist due to immunosuppression and insufficient antigen presentation, necessitating combinatorial strategies to amplify PDIT-driven immunity. Pyroptosis is an inflammatory programmed cell death mediated by gasdermin pore formation. Through the release of pro-inflammatory cytokines and DAMPs, it transforms immunologically "cold" tumors into "hot" microenvironments [17]. Canonical pyroptosis involves caspase-1-mediated cleavage of GSDMD, generating membrane pores and triggering cytokine release. In contrast, PDT specifically activates caspase-3 to cleave GSDME, producing N-terminal fragments without targeting GSDMD [18]. This non-canonical pyroptotic pathway effectively induces tumor cell death while significantly suppressing distant metastasis and postoperative recurrence [19]. Consequently, light-driven induction of pyroptosis represents an emerging strategy to potentiate antitumor immunotherapy [8,12,20]. However, GSDME gene expression is silenced in breast cancer through methylation [21]. Decitabine (DEC), a DNA-demethylating agent, reactivates functional GSDME expression. DEC combined with chemotherapeutics (e.g., paclitaxel [22] or gambogic acid [23]) shows triple-negative breast cancer (TNBC) therapeutic potential. Therefore, we propose a combined strategy using the epigenetic drug DEC with the photosensitizer HPPH to synergistically induce pyroptosis, enhancing the efficacy of breast cancer immunotherapy [24].

Current pyroptosis inducers suffer from instability, unpredictable toxicity, and inadequate efficacy, compounded by poor tumor localization during systemic administration. Nanoparticle delivery systems, including liposomes, solid lipid nanoparticles, and inorganic nanocarriers, can resolve the aforementioned problems [25]. Nevertheless, the off-target toxicity caused by systemic administration remains a significant concern. To overcome this obstacle, local drug delivery has emerged as a highly promising alternative approach. Microneedles (MNs), as minimally invasive transdermal delivery systems, offer self-healing, injectable, and stimuli-responsive properties for precision therapy [26]. For example, Li et al. encapsulated the self-oxygenation and glutathione depletion nanoparticles into a hyaluronic acid microneedle patch, while improving the biosafety and therapeutic efficacy of PDT [27]. Similarly, dissolving MNs co-delivering R848 and aPD-1 reverse TNBC immunosuppression [28]. Synthetic MN patches for localized immunotherapy delivery enhance tumor drug concentrations while minimizing systemic toxicity.

Addressing these challenges, we innovatively designed soluble MNs for co-delivering DEC and HPPH-loaded PLGA-SS-PLGA nanoparticles (HPPH-NP2, N2). Compared to intratumoral or intravenous administration, MNs delivery achieves superior tumor drug accumulation. HPPH-N2 demonstrates enhanced GSH depletion and ROS amplification versus conventional PLGA carriers (HPPH-NP1). This combination effectively activates breast cancer immune microenvironments, and when combined with PD-1 monoclonal antibodies, concurrently treats primary tumors and prevents recurrence, lung metastasis (Graphical Abstract).

## 2 Materials and methods

### 2.1 Materials and reagents

HPPH(≥98 %, H882179), 5-Aza-2′-deoxycytidine (Decitabine, Dec, ≥97 %, A801497)were purchased from Macklin Inc (Shanghai, China). PLGA_50/50_ (MW:5000) and PLGA-SS-PLGA_50/50_ (MW:10,000) were provided by Jinan Daigang Biotechnology Co., Ltd(Jinan, Shandong).CD8(Invitrogen, 14-0808-82) was acquired from Thermo Fisher Scientific Inc. (Massachusetts, USA). Calreticulin Polyclonal antibody (CRT, 10292-1-AP), HMGB1 Polyclonal antibody (HMGB1, 10829-1-AP), DFNA5 Polyclonal antibody (GSDME, 13075-1-AP), and Granzyme B Polyclonal antibody (Granzyme B, 13588-1-AP) were purchased from Proteintech Group, Inc. (Wuhan, China). Cleaved Caspase-3 (Asp175) Antibody #9661 was provided by Cell Signaling Technology, Inc.(Massachusetts, USA). Anti-DFNA5/GSDME antibody-N-terminal (GSDME-N, EPR19859) was purchased from Abcam (Massachusetts, USA). TNF-α(sc-52746) was purchased from Santa Cruz Biotechnology, Inc.(California, USA), Anti -*Anti*-Ki67 Rabbit pAb (Ki-67, GB111499-100) was purchased from Wuhan Servicebio Technology Co., Ltd.(Wuhan, Chine). The following antibody were purchased from BioLegend, Inc. (California, USA): PE anti-mouse CD45 Antibody(147712), FITC anti-mouse CD3 Antibody(100204), PerCP anti-mouse CD4 Antibody(100538), APC anti-mouse CD8a Antibody(100712), PE/Cyanine5 anti-mouse CD8a Antibody(100710), PE/Cyanine7 anti-mouse CD11c Antibody(117318), FITC anti-mouse CD80 Antibody(104706), APC anti-mouse CD86 Antibody(105012), APC anti-mouse/human CD44 Antibody(103012), PE/Cyanine7 anti-mouse CD62L Antibody(104417).

### 2.2 Cell culture

Human breast cancer MDA-MB-231 cells and mouse breast cancer 4T1 cells were purchased from Servicebio (Wuhan) and cultured in high glucose DMEM medium in a CO_2_ incubator at 37°.

### 2.3 Animals

Female Balb/c mice, aged 6–8 weeks, were purchased from BesTest Bio-Tech (Zhuhai, China) and housed in a SPF environment. All animal studies complied with national guidelines for laboratory animal care and were approved by the Ethics Committee of The Third Affiliated Hospital of Guangzhou Medical University (Ethical approval:2024-140).

### 2.4 Preparation and characterization of nanoparticles

The emulsion-solvent evaporation method, with minor revisions, was utilized to prepare the HPPH-loaded nanoparticles. Briefly, PLGA_50/50_ or PLGA_50/50_-SS-PLGA_50/50_ (20 mg) and HPPH (1 mg) were dissolved in 1 mL CH_2_Cl_2_ to form the organic phase and mixed with 5 mL aqueous phase containing 5 % PVA. The mixture was sonicated for 5min (100W), swirled by hand in an ice water bath, resulting in an opalescent dispersion. The relative parameters were fixed as follows, 200W was applied, with 55 cycles of a 3 s sonication and a 3 s pause. This dispersion was stirred at room temperature for 4h under shading to evaporate the residual organic solvent, yielding the raw nanoparticle suspension. Contiguously, the suspensions were sonicated again to obtain a homogenized nanoparticles mixture under the same sonicate parameters above all, designated as HPPH-Np1 (N1), and HPPH-SS-Np2(N2).

The particle size, polydispersity index, and ζ-potential of the liposomes were analyzed using a Zeta Sizer nano-ZS90 (Malvern, UK). The morphology of the liposomes was investigated using transmission electron microscopy (TEM, JEM2100F, Japan) after depositing the samples on a copper grid and staining with 1 % acetic acid glaze. The FTIR spectra of the nanoparticles were obtained using an FTIR spectrophotometer (Bruke TENSOR27, Germany). The differential scanning calorimetry was recorded on a thermal gravimetric analyzer (METTLER TOLEDO TGA 2, Swiss).

### 2.5 In vitro GSH-triggered release of HPPH from nanoparticles

A modified dialysis method was practiced to investigated the drug release behavior of HPPH from nanoparticle formulations N1 and N2 in the presence of varying concentrations of GSH. Concisely, 1 mL of HPPH nanoparticles suspension was enclosed within a dialysis bag (mass cut off 3500), and incubated in 15 mL of an ethanol-phosphate buffer solution (20:80, v/v) containing either 0.01 mM or 10 mM GSH. This setup was maintained under gentle shaking (100 rpm) at 37 °C. Periodically, the medium was collected and replaced with pre-warmed ethanol-phosphate buffer solution containing the corresponding concentrations of GSH. The collected medium was quantified by a UV–vis spectrophotometer(Shimadzu, UV-1780, Japan).

### 2.6 Preparation and characterization of HPPH-loaded microneedles patch

The HPPH-incorporated microneedles were fabricated via a template molding process using a poly(dimethyl siloxane) (PDMS) mold through sequential loading of casting and backing solutions [29,30]. The PDMS mold contains a 20 × 20 array of conical microneedles (MNs), each with a base radius of 300 μm and a height of 800 μm. The array covers an area of 16.7 mm by 16.7 mm with a center-to-center spacing of 700 μm. A casting solution containing HPPH nanoparticles (3 mg/mL) suspended in a 10 % PVA/sucrose matrix (1:1, w/w) was prepared, and 100 μL aliquots were loaded into the mold cavities followed by pressurized air treatment (0.2 MPa, 25 °C, 3 min) to ensure complete cavity filling, with residual surface solution removed using a glass coverslip to achieve uniform tip formation; this casting-drying cycle was repeated five times to optimize drug loading density. Subsequently, a backing solution comprising 10 % PVA and 10 % PVP K30 was applied to the mold surface, and the assembly underwent negative pressure treatment (5 min) to eliminate air bubbles prior to 48h desiccation at ambient temperature in a desiccator. The final MN patches were subsequently removed from the mold using medical adhesive tape and stored in a desiccator at room temperature until needed. **Morphological characterization** was performed using scanning electron microscopy (SEM, GeminSEM 300, Germany) at 5 kV after gold sputtering, confirming needle tip sharpness and homogeneous nanoparticle distribution within the microneedle shafts. **Fluorescence localization analysis** via confocal microscopy (Nikon, A1R + N-STORM, Tokyo, Japan) validated the retention of HPPH-loaded nanoparticles within microneedle tips, with Z-stack imaging.

### 2.7 Uptake of tumor cells and tumor spheres

The cellular internalization kinetics of nanoparticle formulations (N1/N2) were evaluated in both 4T1 monolayer cultures and tumor spheroids using confocal microscopy and flow cytometry. For monolayer studies, cells seeded in confocal dishes or 24-well plates were treated with N1/N2 (0.25 μg/mL HPPH equivalent) for 4h, 8h, 16h, and 24h. Post-treatment, confocal samples were fixed and nuclear-stained with DAPI for imaging, while flow cytometry samples were trypsinized and analyzed for intracellular HPPH fluorescence (PerCP-Cy5.5 channel) [6].

Tumor spheroids were established by culturing 1 × 10^4^ 4T1 cells/well in 2 % low-melt agarose-coated 96-well plates for 5 days. Spheroids underwent identical nanoparticle treatment conditions (0.25 μg/mL HPPH, 4–24 h), followed by DAPI staining and z-axis confocal scanning (Nikon A1R + N-STORM, 15 μm slice thickness) to assess depth-dependent nanoparticle penetration.

### 2.8 Cytotoxicity assay

4T1 cells or MDA-MB-231 cells were seeded at a density of 5 × 10^3^ cells/well in 96-well. The cells were treated with various concentrations of HPPH-loaded nanomedicine (10, 5, 2.5, 1.3, 0.6, 0.3, 0.2, 0.1 μg/mL), and the medium was incubated with the cells for 24 h before being replaced with fresh medium. Subsequently, the cells were exposed to a 660 nm laser (100 mW/cm^2^, 3 min), and then incubated for an additional 24h.

In addition, the cytotoxicity effects of DEC on breast cancer cells were investigated using the drug-containing medium with different concentrations of (1.6, 3.1, 6.3, 12.5, 25, 50, 100, 200, 400 μM) over a 48-h treatment period. The cytotoxicity was detected by the MTT assay, and the absorbance was measured at 490 nm on an enzyme labeling instrument to calculate the cell viability.

### 2.9 ROS generation analysis in monolayer and spheroid models

ROS induction by HPPH-loaded nanoparticles was systematically evaluated across concentration gradients and treatment modalities. 4T1 cells seeded in 24-well plates or confocal dishes were treated with HPPH (0.125 μg/mL,0.25 μg/mL, 0.5 μg/mL) for 24 h, followed by 660 nm laser irradiation (100 mW/cm^2^, 3 min). Six experimental groups (PBS, PBS + Laser, N1, N2, N1+Laser, N2+Laser) were established at 0.25 μg/mL HPPH concentration. Post-treatment, cells were incubated with 10 μM DCFH-DA for 1 h, with ROS-associated green fluorescence quantified via flow cytometry and confocal imaging (Nikon, A1R + N-STORM, Tokyo, Japan). For tumor spheroids, identical treatment parameters were applied. Z-axis confocal scanning (15 μm slice thickness) enabled three-dimensional visualization of ROS distribution within spheroids.

### 2.10 Detection of GSH consumption

To assess the GSH levels of 4T1 cells after various treatments, 4T1 cells inoculated in 6-well plates were treated with 0.25 μg/mL of either N1 or N2 for 6h. Eventually, the cells were harvested, and the GSH consumption was detected by the GSH/GSSG detection kit according to the instructions provided by the manufacture (G4304, Servicebio, China).

### 2.11 Detection of cellular pyroptosis

The 4T1 cells were spread on a 24-well plate for the experiment, which was divided into PBS, DEC, N2+Laser, DEC + N2+Laser. The concentration of DEC was 20 μM for 12h, and the concentration of HPPH was 0.25 μg/mL for 24h. After replacing the new medium, the cells were exposed to a 660 nm laser (100 mW/cm^2^, 3 min), and continued to be cultivated for 6 h, the formation of pyrogenic bodies was observed by inverted microscope(Nikon, ECLIPSE Ti-S, Tokyo, Japan).

### 2.12 Cell apoptosis assay

The 4T1 cells were spread into 12-well plates. And the experiment was divided into 6 groups of PBS, PBS + Laser, N1, N2, N1+Laser, N2+Laser, and the concentration of HPPH was 0.25 μg/mL. After incubation for 24h, the medium was replaced, and the cells were irradiated by 660 nm laser (100 mW/cm^2^, 3 min). The incubation continued for an additional 24h to examine the effect on apoptosis in the 4T1 cells. Secondly, the experiment was divided into PBS, DEC, N2+Laser, DEC + N2+Laser. DEC was administered at a concentration of 20 μM for 12 h, and HPPH was administered at a concentration of 0.25 μg/mL for 24 h. After these treatments, the medium was replaced, and the cells were irradiated with a 660 nm laser (100 mW/cm^2^, 3 min).

For apoptosis detection, the cells were stained according to the protocols supplied with an apoptosis detection kit (Beyotime Biotechnology, Shanghai). Specifically, Annexin V-FITC and Propidium Iodide (PI) staining was conducted for 15 min. Apoptotic cells were then quantified using flow cytometry (Thermo Fisher Scientific).

### 2.13 Live/dead staining

The 4T1 cells were seeded into 24-well plates and assigned to four experimental groups: PBS, DEC (20 μM, 12 h), N2+Laser (0.25 μg/mL HPPH, 24 h + 660 nm laser [100 mW/cm^2^, 3 min]), and DEC + N2+Laser. Following treatment and 24 h post-irradiation culture, cell viability was assessed using Calcein-AM/PI dual staining. Cells were washed twice with assay buffer (2 min/wash), sequentially stained with Calcein-AM (2 μM, 37 °C, 25 min) and PI (5 μM, 5 min), then imaged via fluorescence microscopy (Nikon, ECLIPSE Ti-S, Tokyo, Japan). For tumor spheroids cultured in 96-well plates, identical treatment parameters (DEC concentration/duration, HPPH dose, laser conditions) were applied. Post-staining, spheroid viability was analyzed through z-axis confocal scanning (15 μm slice thickness) to quantify depth-dependent cytotoxicity.

### 2.14 Western blotting

After 4T1 cells were administered and treated, cell lysate was added to extract proteins. The protein concentration in the lysates was measured using a BCA Protein Assay Kit. Proteins were then separated by SDS-PAGE electrophoresis and subsequently transferred onto a membrane. To block non-specific binding, the membrane was incubated with 5 % milk for 1 h. After blocking, the membrane was incubated overnight at 4 °C with primary antibodies targeted against Caspase3, Cleaved Caspase3, GSDME, and GSDME-N, each diluted 1:1000. Following the overnight incubation, the membrane was washed to remove any unbound antibodies. A secondary antibody, diluted 1:2000, was then applied and left to incubate for 1 h at room temperature. Finally, the membrane was developed using an enhanced chemiluminescence (ECL) detection system to visualize the protein bands.

### 2.15 Immunocytochemistry staining

4T1 cells were spread on the confocal dish and divided into PBS, DEC, N2+Laser, DEC + N2+Laser. After treatment, the cells were fixed with 4 % paraformaldehyde for 15 min and washed twice with PBS. A 5 % BSA solution was used for blocking at room temperature for 1h. and incubated with either HMGB1 (1:100) diluted with 0.1 %Triton-100 PBS or CRT(1:100) diluted with PBS overnight. On the second day, the cells were washed three times with PBS, then incubated with either DyLight 649(1:200) for HMGB1 or DyLight 488(1:200) for CRT at room temperature. Nuclei were stained with DAPI for 3 min, and then photographs were taken with a confocal microscope (Nikon, A1R + N-STORM, Tokyo, Japan).

### 2.16 ATP and LDH assay

4T1 cells were cultured in 6-well (for ATP assay)/96-well plates (for LDH assay) and treated with PBS, DEC, NP2+Laser, DEC + NP2+Laser. Following the treatments, the cell culture supernatants were harvested to measure the ATP or LDH content using the ATP (S0026, Beyotime, China) or LDH (C0016, Beyotime, China) detection kit according to the manufacturer's instructions.

### 2.17 Elisa assay

4T1 cells were cultured in 24-well plates and subjected to treatments with PBS, DEC, NP2+Laser, DEC + NP2+Laser. Subsequently, the cell culture supernatants were collected to measure the released HMGB1, IL-1β (MM-0040M2, Mmbio, China), IL-18 (MM-0169M2, Mmbio, China) content from the cell using an ELISA kit according to the manufacturer's instructions.

### 2.18 Primary extraction of BMDC co-cultured with tumor cells

Bone marrow-derived dendritic cells (BMDCs) were isolated from mice and cultured for 6 days in RPMI-1640 medium containing 10 % FBS, recombinant murine GM-CSF (20 ng/mL), and IL-4 (10 ng/mL). DCs purity was verified by flow cytometry (CD11c^+^ ≥85 %) prior to experimentation.

4T1 cells were treated under four conditions: PBS, DEC (20 μM, 12 h), N2+Laser (0.25 μg/mL HPPH, 24 h + 660 nm laser [100 mW/cm^2^, 3 min]), and DEC + N2+Laser. Post-treatment cells were co-cultured with BMDCs at 1:5 ratio (tumor cell: DC) for 24 h. LPS-stimulated BMDCs (1 μg/mL, 24 h) served as maturation-positive controls. Mature DCs were identified via triple staining with anti-CD11c-PE/Cyanine7/CD80-FITC/CD86-APC antibodies (30 min, 4 °C) and analyzed by flow cytometry.

### 2.19 In vivo imaging

An orthotopic tumor model was established using 4T1 cells transplanted into Balb/c mice, where the tumors were allowed to grow until they reached a volume of 300–500 mm^3^. N1 or N2 was administered by intratumoral injection, tail vein injection, and microneedle administration, and the concentration of HPPH was 0.25 μg/mL. Three days post-administration, the mice were euthanized, and organs such as the tumor, heart, liver, spleen, lung, and kidney were removed. The fluorescence intensity of the isolated tissues was observed by in vivo imaging (PerkinElmer, Waltham, MA, USA) to assess the biodistribution and accumulation of the nanoparticles.

### 2.20 In vivo therapeutic evaluation and drug administration

Female BALB/c mice bearing orthotopic 4T1 mammary tumors (100–200 mm^3^) were divided into six treatment cohorts: Saline, DEC monotherapy (1 mg/kg, intratumoral), N1+Laser (0.5 mg/kg HPPH + 660 nm laser irradiation [100 mW/cm^2^, 10 min]), N2+Laser, N1+Laser + DEC, and N2+Laser + DEC, with all intratumoral administrations performed weekly. Tumor volumes (L × W^2^/2) and body weights were monitored biweekly until endpoint, followed by tumor excision, weighing, and H&E histological analysis (Nikon Ni-U).

To evaluate delivery route efficacy, a separate cohort received one of four treatments: saline, intratumoral N2+Laser + DEC, intravenous N2+Laser + DEC, or microneedle-mediated N2+Laser + DEC, using identical dosage and monitoring protocols to the study mentioned above.

### 2.21 In vivo combined immunotherapy in a recurrent tumor model

Orthotopic 4T1 tumors in BALB/c mice were surgically resected upon reaching ∼300 mm^3^, followed by initiation of adjuvant therapy 1 day post-operation [28,31]. Four treatment regimens were evaluated: saline, microneedle patch (MNs) delivering N2 nanoparticles (0.5 mg/kg HPPH) and decitabine (1 mg/kg), intraperitoneal PD-1 monoclonal antibody (100 μg/mouse), and combinatorial MNs + PD-1 mAb. The MN patch was positioned at the resection site and activated by 660 nm laser irradiation (100 mW/cm^2^, 10 min) at 12 h and 36 h post-application, while PD-1 mAb was administered via i.p. injection.

Tumor recurrence kinetics were tracked through biweekly measurements of resection site dimensions (volume = length × width^2^/2) and body weight changes. Terminal evaluation involved excision of primary tumors and major organs (heart, liver, spleen, kidneys) for histopathological analysis via H&E staining. Lung metastases were quantified using Digital pathology slide scanner (Leica Aperio CS2, Germany).

### 2.22 Immunofluorescence staining

Tumor tissue sections were deparaffinized and antigen-retrieved, followed by blocking with 10 % goat serum at room temperature for 1 h. Primary antibodies including Ki67, CRT, HMGB1, CD8, Foxp3, CD163, Cleaved caspase3, GSDME-N, TNF-α, granzyme B, was incubated overnight in a wet box at 4 °C. The sections were then washed three times with PBS for 5 min each. Fluorescent secondary antibody DyLight649 or DyLight488 (1:200) were applied for 1h followed by three additional 5 min PBS washes, and then dropwise added with anti-fluorescent bursting agent containing DAPI counterstain the nuclei. Finally, the stained sections were photographed and observed by confocal microscope (Nikon, A1R + N-STORM, Tokyo, Japan).

### 2.23 TUNEL staining

Paraffin sections were deparaffinized, rehydrated, and treated with proteinase K for antigen retrieval. TUNEL staining was performed using FITC-streptavidin (1:9 in buffer, 37 °C, 30 min, dark). After DAPI counterstaining and PBS washes, slides were imaged by confocal microscopy (Nikon, A1R + N-STORM, Tokyo, Japan).

### 2.24 In vivo flow study

A portion of freshly collected tumor, and tumor draining lymph node tissues were used for protease digestion, homogenized by rinsing with PBS, and passed through a 70 μm nylon mesh to obtain a single cell suspension. The isolated cells were stained with fluorescent direct labeling antibodies for 30 min. Subsequently, flow cytometry analysis (Thermo Fisher Scientific) was employed to quantify the number of different cell types in the tissues. For mature dendritic cells were identified and labeled with CD45^+^CD11c^+^CD80^+^CD86^+^, T cells were labeled with CD45^+^ CD3^+^ CD4^+^ CD8^+^.Memory T cells in Spleens were detected by flow cytometry, CD4T memory cells were labeled with CD3^+^CD4^+^CD44^+^CD62L^+^, CD8T memory cells were labeled with CD3^+^CD8^+^CD44^+^CD62L^+^.

### 2.25 Statistical analysis

All quantitative data are presented as means ± standard deviation (SD). The data were analyzed using GraphPad Prism Software. Statistical significance was determined using a two-tailed Student's t-test for two groups and one-way analysis of variance (ANOVA) for multiple groups. Significant differences between groups were indicated by ∗p < 0.05, ∗∗p < 0.01, and ∗∗∗p < 0.001, respectively.

## 3 Results

### 3.1 Characterization of co-loaded decitabine and HPPH-ss-N2 soluble microneedle patch

**Fig. 1:**
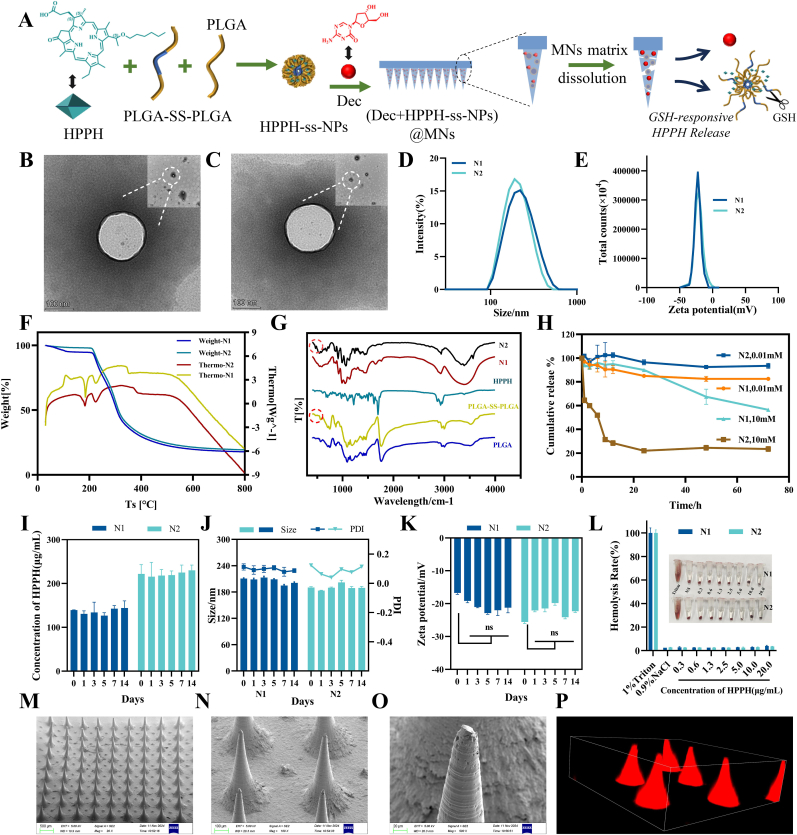
Characterization of HPPH loaded nanoparticles and microneedles patch. (A) N2s synthesized via disulfide-modified PLGA, were co-loaded with DEC into PVA-based microneedles using micromolding. Transmission electron microscopy images of N1 (B) and N2 (C). Size distribution image (D) and zeta potential diagram (E) of N1 and N2. (F) Thermogravimetric analysis spectrum of both N1 and N2. (G)FTIR spectrum of nanoparticles. (H)In vitro release kinetics of HPPH in PBS at 37 °C under different concentration GSH from nanoparticles. The content (I), particle size (J) and zeta potential (K) stability assessments of nanoparticles. (L) Hemolysis test results for the nanoparticles. (M,N,O) Scanning electron microscopy images of (DEC+HPPH-ss-N2) microneedle patches. (P) CLSM 3D reconstruction images of (DEC+HPPH-ss-N2) microneedle patches.

Redox-responsive HPPH-ss-NPs were prepared by encapsulating HPPH into disulfide-bridged PLGA nanoparticles. DEC and HPPH-ss-NPs were then blended with a PVA matrix, centrifuged into a PDMS mold, and dried to form dissolvable microneedles, achieving dual-drug co-loading with tumor-specific release(Fig. 1A). TEM revealed monodisperse spherical morphologies for both N1 and N2 nanoparticles, with dry-state diameters of 140 nm and 145 nm, respectively (Fig. 1B–C). Dynamic light scattering measurements demonstrated hydrated particle sizes of 187.5 ± 0.68 nm (N1) and 201.8 ± 5.18 nm (N2) (Fig. 1D), consistent with polymeric nanoparticle swelling behavior. Both formulations exhibited negative zeta potentials (−24.1 ± 0.5 mV for N1, -22.9 ± 0.4 mV for N2) ensuring colloidal stability (Fig. 1E). The DSC images demonstrated that both two nanoparticles possessed similar curve change (Fig. 1F). Fourier-transform infrared spectroscopy confirmed successful HPPH encapsulation through characteristic N-H (3393 cm^−1^) and C-H (2920 cm^−1^) stretching vibrations (Fig. 1G). Notably, N2 displayed a distinct disulfide bond absorption peak at 469 cm^−1^, mirroring the PLGA-SS-PLGA polymer profile (Fig. 1G).

Under physiological GSH levels (10 μM), both nanoparticles maintained sustained HPPH release. However, N2 exhibited tumor microenvironment-triggered release in 10 mM GSH conditions, achieving 76.5 ± 1.5 % cumulative release within 72 h, which increase over N1 (Fig. 1H). This differential release kinetics confirmed the redox-sensitive disulfide bond cleavage mechanism in N2 (Fig. 1I). Additionally, Fig. S1 reveals morphological changes in N2 following glutathione exposure through TEM. Both formulations retained >90 % HPPH payload after 14-day storage at 4 °C (Fig. 1J), with negligible changes in particle size (Fig. 1I) and surface charge (Fig. 1K). Hemolysis assays confirmed blood compatibility, showing <2 % hemolysis across 0.2–20 μg/mL HPPH concentrations(Fig. 1L).

SEM confirmed the microneedle array's structural integrity, displaying uniform conical needles (Fig. 1M) with symmetrical tip architecture (Fig. 1N and O). Successful HPPH nanoparticles incorporation was visually verified by distinct red coloration at needle tips (Fig. 1P).

### 3.2 Cellular uptake and ROS generation under light irradiation

**Fig. 2:**
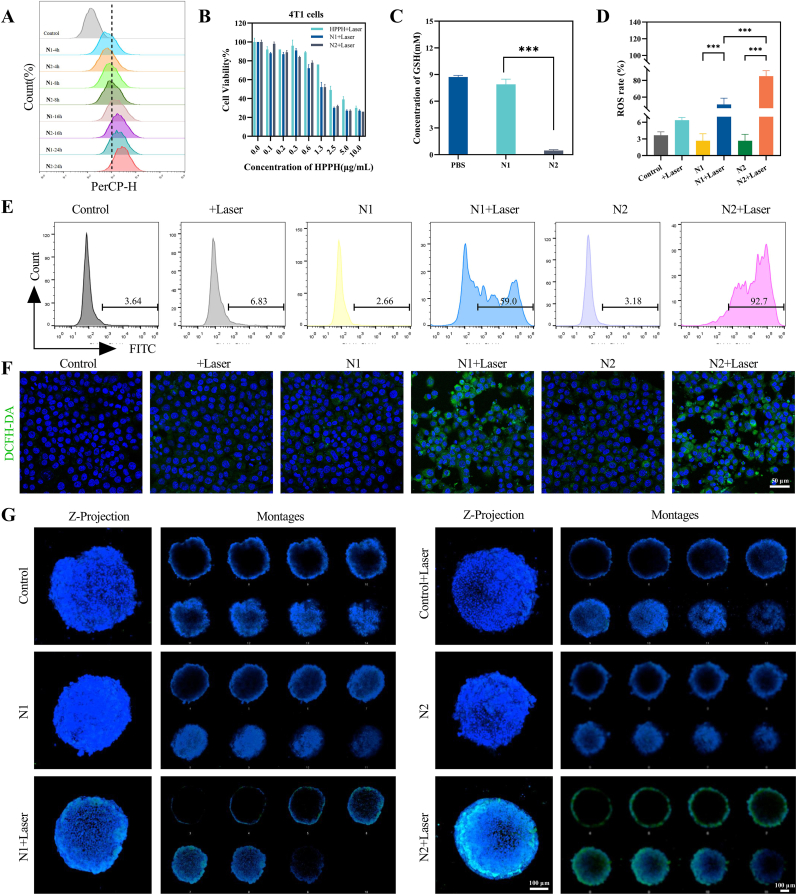
Cellular uptake and ROS generation of HPPH-loaded nanoparticles in 4T1 cells and 3D tumor spheres. (A) Flow cytometry detection of nanoparticle uptake by 4T1 cells. (B) Cytotoxicity results for HPPH and its formulations against 4T1 cells. (C) Effect of nanoparticles on glutathione levels in 4T1 cells. (D, E) Flow cytometry detection of ROS levels in 4T1 cells and statistical analysis. (F) CLSM images of ROS production in 4T1 cells treated with different formulations. (G) CLSM images of ROS production in 4T1 cell 3D spheroids treated with different nanoparticle formulations of HPPH.

Flow cytometry and confocal imaging revealed progressive intracellular accumulation of HPPH-loaded nanoparticles in 4T1 cells, with N2 exhibiting 1.18-fold higher fluorescence intensity than N1 at 24 h (Fig. 2A, S2). This uptake superiority persisted in 3D tumor spheres, where N2 demonstrated enhanced penetration depth (Fig. S3), suggesting redox-responsive disulfide bonds facilitated endosomal escape. Dose-response analysis demonstrated distinct cytotoxic profiles between formulations, in murine 4T1 cells, HPPH exhibited an IC_50_ of 3.37±0.19 μg/mL, while nanoparticle-encapsulated forms showed enhanced potency with N1 (1.92±0.09 μg/mL) and N2 (1.63±0.07 μg/mL)(Fig. 2B). This trend persisted in human MDA-MB-231 cells, where IC_50_ values were 4.66±0.26 μg/mL (free HPPH), 2.58±0.10 μg/mL (N1), and 2.12±0.10 μg/mL (N2) (Fig. S4).

Optimal ROS induction occurred at 0.25 μg/mL HPPH (Fig. S5), balancing efficacy against cell death-induced signal attenuation at higher doses. N2's GSH-depleting capacity (Fig. 2C) synergized with PDT to amplify oxidative stress. Among the cell populations, 92.7 % of the N2+Laser treated cells showed positive DCFH-DA staining, in contrast to 59.0 % of the N1+Laser treated cells(Fig. 2D and E). Laser irradiation-activated photosensitizers generated ROS in both 2D 4T1 monolayers and 3D tumor spheres, with N2+Laser exhibiting stronger green fluorescence intensity (indicative of ROS accumulation) compared to N1+Laser (Fig. 2F). Spatial analysis confirmed enhanced ROS propagation throughout 3D tumor spheres in the N2+Laser group (Fig. 2G).

**Fig. 3:**
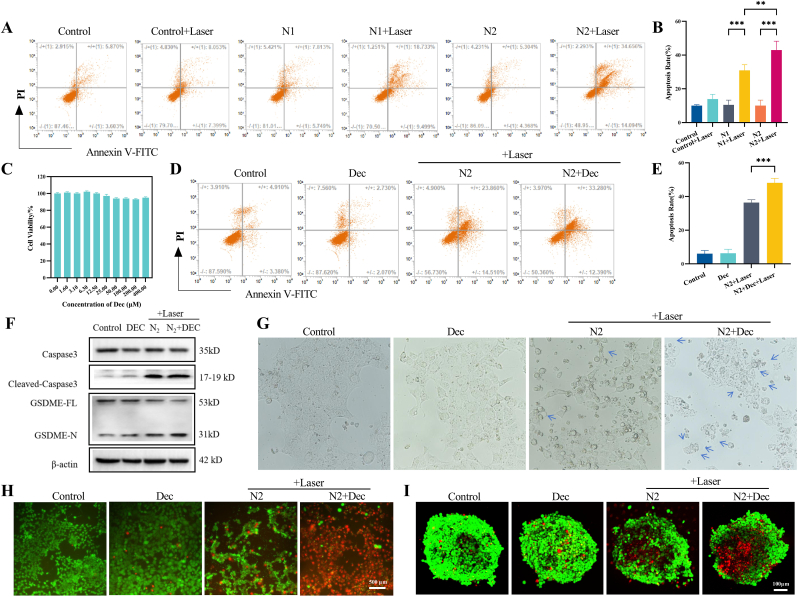
Photodynamic-triggered conversion of apoptosis to pyroptosis in 4T1 cells by combined HPPH-loaded nanoparticles and DEC. (A, B) Flow cytometry results and statistical analysis of the effects of different nanoparticles on apoptosis in 4T1 cells. (C) Cytotoxicity effects of Dec on the viability of 4T1 cells. (D, E) Flow cytometry results and statistical analysis of the effects of HPPH-ss-N2 combined with Dec on apoptosis in 4T1 cells. (F) Western blot images of apoptosis-related proteins in 4T1 cells treated with HPPH-ss-N2 combined with Dec. (G) Images of pyroptotic bodies in 4T1 cells treated with HPPH-ss-N2 combined with Dec. (H,I) Live and dead assay of 4T1 cells and 4T1 tumor spheroids treated with HPPH-ss-N2 combined with Dec.

Consistent with these findings, cleaved caspase-3 expression displayed concentration-dependent elevation (Fig. S6A). Comparative apoptosis assays revealed N2+Laser induced 42.98 ± 5.24 % cell death versus 30.90 ± 3.45 % for N1+Laser (Fig. 3A–B), with corresponding cleaved caspase-3 levels confirming N2's superior apoptotic activation (Fig. S6B). These data establish N2's dual advantage, enhanced tumor cell targeting through GSH depletion and amplified apoptotic signaling via ROS potentiation.

### 3.3 Photodynamic-triggered conversion of apoptosis to pyroptosis in 4T1 cells

Decitabine pretreatment induced negligible cytotoxicity (Fig. 3C) while restoring tumor-suppressed GSDME expression through epigenetic modulation (Fig. S7). Although decitabine alone did not increase apoptosis rates, its combination DEC with NP2+Laser elevated apoptosis-positive cells to 48.13 ± 2.70 %, compared to 36.44 ± 1.63 % in NP2+Laser group (Fig. 3D–E).

Mechanistically, the expression of cleaved caspase-3 was upregulated in the combined treatment group, and cleaved caspase-3-mediated GSDME cleavage generated functional N-terminal fragments (GSDME-N) that formed plasma membrane pores (Fig. 3F, S8A-C), evidenced by characteristic pyroptotic body formation in the DEC + N2+Laser group (Fig. 3G). Live/dead staining revealed cytotoxicity profiles between 2D and 3D models under combinatorial therapy. In 2D monolayers, the DEC + N2+Laser group exhibited near-complete viability loss characterized by PI-dominant staining, contrasting with sporadic dead cells in monotherapy groups(Fig. 3H). The 3D tumor spheres exhibited extensive cell death in the central region while retaining peripheral viability (Fig. 3I). These data demonstrate decitabine's critical role in converting PDT-induced apoptosis into pyroptosis-dominated cell death.

### 3.4 Synergistic activation of ICD and dendritic cell maturation by HPPH-ss-N2/DEC combinatorial therapy

**Fig. 4:**
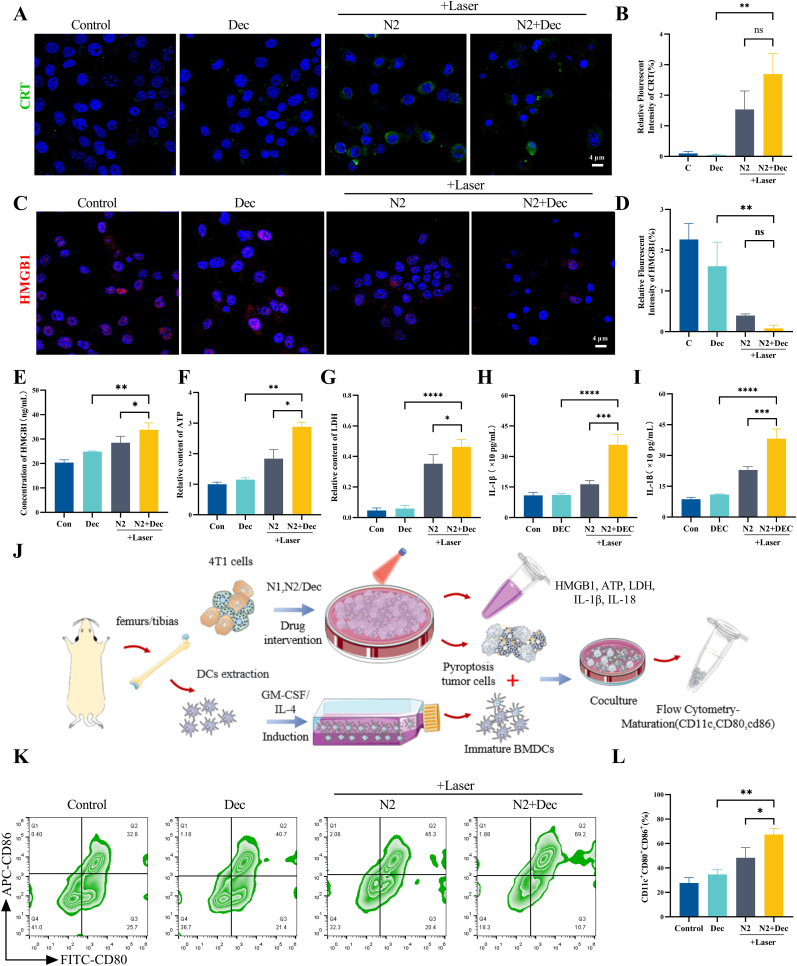
Dendritic cell maturation induced by PDT-mediated immunogenic cell death and pyroptosis. Confocal microscopy images and their statistical analysis of CRT (A, B) and HMGB1 (C, D) expression in 4T1 cells after treatment with HPPH-ss-N2 combined with Dec. Levels of HMGB1 (E), ATP (F), LDH (G), IL-1β(H), and IL-18(I) released after treating 4T1 cells with HPPH-ss-N2 combined with Dec. (J) Schematic diagram of the in vitro experiment for inducing DCs maturation. (K, L) Flow cytometry analysis and statistical data of mature DC cell proportions.

The combined regimen of DEC and HPPH-ss-N2 nanoparticles under laser irradiation (DEC + N2+Laser) synergistically enhanced both ICD and pyroptotic signaling in 4T1 tumor cells. Confocal imaging revealed robust translocation of CRT to the cell membrane in the DEC + N2+Laser group, evidenced by intensified green fluorescence along cellular peripheries compared to monotherapy groups (Fig. 4A–B). Concurrently, nuclear-to-cytoplasmic re-localization of HMGB1 was observed, with immunofluorescence showing a reduction in nuclear HMGB1 retention and a 1.18-fold increase in extracellular HMGB1 release compared to N2+Laser alone (Fig. 4C–E). This coordinated DAMPs release was further amplified by elevated ATP secretion (Fig. 4F) and LDH surge (Fig. 4G), while stimulating secretion of pro-inflammatory cytokines IL-1β (Fig. 4H) and IL-18 (Fig. 4I), confirming dual activation of ICD and pyroptosis.

The immunogenic effects translated to DCs maturation in co-culture models. Bone marrow-derived DCs exposed to DEC + N2+Laser-treated tumor cells exhibited a maturation rate of 67.33 ± 5.03 % (CD11c^+^CD80^+^CD86^+^), significantly surpassing both N2+Laser (48.33 ± 8.39 %) and DEC monotherapy (34.67 ± 4.04 %) groups (Fig. 4H–J). The combined therapy effectively activates ICD and DCs maturation, transforming immunosuppressive tumors into immunoreactive environments that prime systemic anti-tumor immunity.

### 3.5 In vivo antitumor efficacy of HPPH-ss-N2 combined with decitabine in breast cancer

**Fig. 5:**
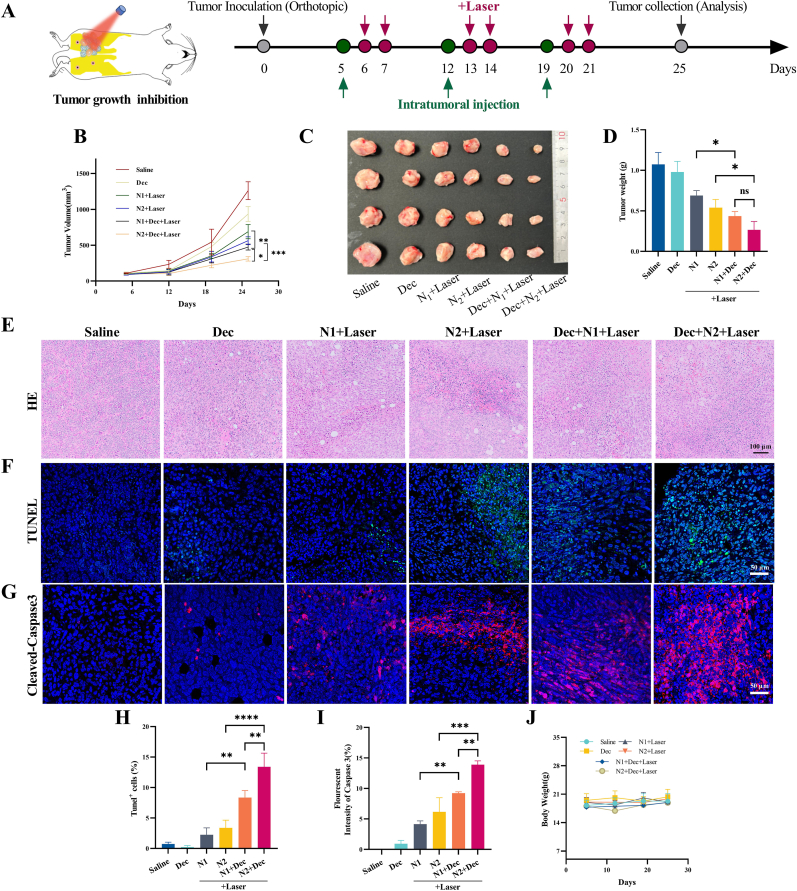
In vivo antitumor efficacy of HPPH-ss-N2 combined with decitabine in 4T1 breast cancer mouse model. (A) Schematic representation of the treatment regimen. (B) Tumor volume curve diagram measured throughout the study. (C,D) Photograph of the ex vivo tumor and tumor weights measured at the endpoint of the efficacy studies. (E) Light photograph of the tumor sections stained by HE, (F, H) TUNEL, (G, I) Cleaved-Caspase 3 and corresponding data analyze. (J) Body weight changes of 4T1 tumor bear mice.

Orthotopic 4T1 tumor-bearing mice received weekly intratumoral injections over three weeks (Fig. 5A). Saline-treated tumors exhibited rapid progression (1260.73 ± 124.00 mm^3^ final volume, 1.07 ± 0.15 g weight), while combinatorial DEC + N2+Laser therapy achieved maximal suppression (310.06 ± 32.30 mm^3^, 0.27 ± 0.10 g, 74.76 % inhibition rate by weight). The redox-responsive N2+Laser group showed enhanced tumor control compared to N1+Laser, with tumor weights of 0.54 ± 0.10 g (49.65 % inhibition) versus 0.69 ± 0.06 g (35.51 %). DEC + N2+Laser combination therapy demonstrated clear therapeutic advantage over individual monotherapies, highlighting the synergistic potential of PDT and epigenetic modulation(Fig. 5B–D), with a favorable safety profile evidenced by stable mouse body weight throughout treatment (Fig. 5J).

Histopathological analysis revealed extensive tumor necrosis and reduced Ki67^+^ proliferative cells in treated groups (Fig. 5E, S9A-B). TUNEL assays showed combinatorial therapy induced 13.40 ± 2.25 % apoptosis (Fig. 5F–H), paralleled by cleaved caspase-3 upregulation (Fig. 5G–I). Crucially, caspase-3 activation triggered pyroptosis via GSDME-N pore formation, evidenced by higher GSDME-N expression in DEC + N2+Laser tumors versus controls (Fig. S10A–B).

Comparative biodistribution analysis revealed intravenous (i.v.) HPPH administration accumulated in spleen and liver tissues, while localized intratumoral (i.t.) and microneedle (MN) delivery achieved preferential tumor enrichment with minimal off-target distribution, demonstrating reduced systemic toxicity (Fig. S11A). Tumor fluorescence intensity was comparable between MN and i.t. groups, both markedly exceeding i.v. levels (Fig. S11B), suggesting equivalent intratumoral drug retention. This tumor-targeting advantage translated to potent therapeutic outcomes, yielding tumor weights of 0.23 ± 0.08 g (MN), 0.27 ± 0.09 g (i.t.), 0.37 ± 0.03 g (i.v.) and 0.74 ± 0.06 g (saline) (Fig. S12A–C). Histopathological analysis confirmed MN-induced tumor necrosis (Fig. S12E), while stable body weights verified safety (Fig. S12D). This localized strategy circumvents photosensitizer accumulation in healthy tissues, addressing a key translational barrier.

### 3.6 In vivo TME reprogramming and immune activation in breast cancer

**Fig. 6:**
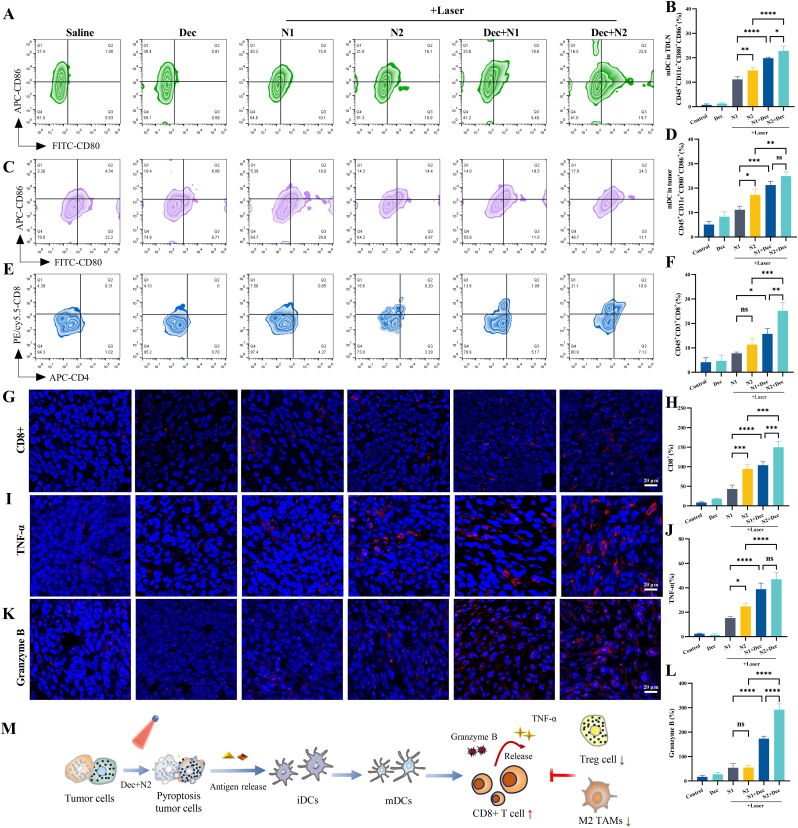
Remodeling of the tumor immune microenvironment by HPPH-ss-N2/DEC combinatorial therapy in 4T1 murine breast tumors. (A, B)The flow cytometry results and statistical analysis of CD80^+^CD86^+^ cells in mouse lymph node tissues. Flow cytometry results and statistical analysis for CD80^+^CD86^+^ cells (C, D) and CD4^+^CD8^+^ cells (E, F) in mouse tumor tissues. Immunofluorescence CLSM images and their statistical analysis of CD8^+^ cells (G, H), TNF-α (I, J), and Granzyme B (K, L) in mouse tumor tissue sections. (M) Schematic diagram illustrating the impact of nanoparticle formulations combined with Dec on the tumor immune microenvironment.

The DEC+N2+Laser combination therapy triggered dual ICD and pyroptosis in 4T1 tumors, as evidenced by spatial redistribution of DAMPs. Confocal imaging revealed calreticulin translocation to the plasma membrane (Fig. S13A–B) and HMGB1 nuclear-to-extracellular re-localization (Fig. S14A–B) in N2+Laser-treated tumors. DEC monotherapy failed to alter HMGB1 localization, whereas DEC + N2+Laser nearly abolished nuclear HMGB1, demonstrating epigenetic priming enhances DAMPs exposure. This DAMPs release cascade drove systemic DCs activation. In tumor-draining lymph nodes (TDLNs), DEC + N2+Laser increased CD45^+^CD11c^+^CD80^+^CD86^+^ mature DCs to 22.80 ± 2.00 %, higher than DEC alone (1.31 ± 0.34 %) and N2+Laser monotherapy (14.08 ± 1.31 %) (Fig. 6A–B). Tumor-infiltrating DCs showed parallel maturation trends (24.90 ± 1.78 % vs. 17.20 ± 2.44 % for N2+Laser), indicating local and systemic immune activation (Fig. 6C–D).

Mature DCs primed adaptive immunity, elevating tumor-infiltrating CD8^+^ T cells to 25.10 ± 3.47 % in DEC + N2+Laser tumors (vs. 4.12 ± 1.80 % in saline) without altering CD4^+^ populations (Fig. 6E–F). Immunofluorescence revealed enhanced intratumoral CD8^+^ T cell infiltration, with pronounced penetration into tumor core regions in DEC + N2+Laser-treated mice (Fig. 6G–H), accompanied by increased Granzyme B and TNF-α expression relative to saline (Fig. 6I–L). Concurrently, DEC+N2+Laser reduced regulatory T cells (Foxp3^+^ cells) and M2 macrophages (CD163^+^ cells) (Figs. S15–S16), reshaping the immunosuppressive landscape. Integrative analysis (Fig. 6M) illustrates how light-controlled pyroptosis converts "cold" tumors into immunogenic niches through coordinated DAMPs release, DCs-mediated T cell priming, and immunosuppressive cell clearance, a tripartite mechanism enabling sustained anti-tumor immunity.

### 3.7 Combination therapy of (HPPH-ss-N2+DEC) MNs with PD-1 Blockage in breast cancer

**Fig. 7:**
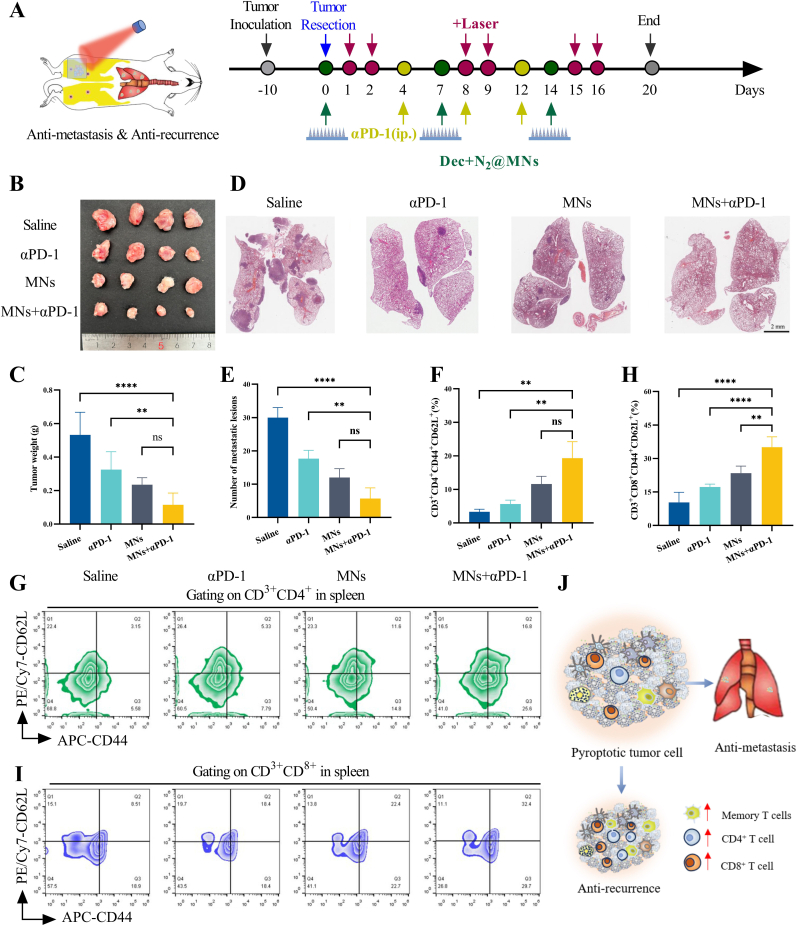
Therapeutic effects of HPPH-ss-N2/DEC-loaded microneedle patches on tumor recurrence and lung metastasis were evaluated in a 4T1 breast cancer mouse model. (A) Schematic diagram of the experiment using microneedle patches for treating 4T1 breast cancer recurrence and lung metastasis models in mice. (B,C) Ex vivo images of the in situ recurrent tumors and their weights. (D,E) Images of metastatic lesions and corresponding quantitative analysis of the number of metastatic lesions. Flow cytometry results and statistical analysis of the proportion of CD44^+^CD62L^+^ cells within CD3^+^CD4^+^ (F, G) and CD3^+^CD8^+^ (H, I) cells in mouse spleen tissues. (J) Schematic diagram illustrating the impact of (DEC + HPPH-ss-N2)@MNs combined with anti-PD-1 on the recurrence and lung metastasis.

The therapeutic regimen integrating microneedle-mediated (HPPH-ss-N2+DEC) MNs and systemic PD-1 blockade demonstrated potent control over both local recurrence and systemic metastasis in a postsurgical breast cancer model (Fig. 7A). Following tumor resection at 300 mm^3^, combinatorial therapy achieved 77 % suppression of local tumor regrowth (0.12 ± 0.07 g), contrasting with extensive recurrence in saline-treated controls (0.53 ± 0.14 g) (Fig. 7B–C). Histopathological analysis revealed most tumor cell clearance in combination-treated lesions, with minimal residual viable cells versus partial necrosis in monotherapy groups (Fig. S17). Critically, this approach elicited systemic abscopal effects, nearly eliminating lung metastases in combination-treated mice compared to metastatic nodules in αPD-1 monotherapy cohorts (Fig. 7D–E, S18).

Long-term immunological memory was established through central effector memory T cells (T_em_) expansion in splenic reservoirs. Combinatorial therapy elevated CD4^+^ T_em_ populations to 19.30 ± 4.95 %, exceeding the 11.61 ± 2.29 % observed with (HPPH-ss-N2+DEC)MNs alone (Fig. 7F–G). Similarly, CD8^+^ T_em_ cells reached 35.03 ± 4.65 % under combinatorial treatment, outperforming both single-agent regimens (23.47 ± 3.14 % for MNs; 17.27 ± 1.27 % for αPD-1) (Fig. 7H–I).

Mechanistically, localized pyroptosis/ICD activation via MNs synergized with PD-1 blockade to overcome stromal immunosuppression, enabling cytotoxic T cell infiltration into residual tumor niches while generating systemic immune memory(Fig. 7J). The regimen maintained favorable safety profiles, with no observed hepatorenal toxicity or weight loss across treatment groups (Figs. S19 and S20).

## 4 Discussion

Microneedle-mediated transdermal drug delivery offers a minimally invasive approach with localized therapeutic effects and reduced systemic toxicity, particularly advantageous for treating superficial malignancies [26,32]. This study advances the field by engineering a dissolvable MNs patch with three key innovations, integration of GSH-depleting nanoparticles to improve HPPH water solubility and PDT efficacy, synergistic enhancement of PDT through decitabine-induced pyroptosis, and combinatorial use with PD-1 monoclonal antibodies to suppress breast cancer progression, recurrence, and metastasis. These design principles address critical limitations in conventional PDT while capitalizing on immunomodulatory synergy.

Pyroptosis is a lytic programmed cell death pathway distinct from apoptosis that bypasses apoptotic resistance and triggers tumor-specific immune responses [12,17,33]. Apoptosis is primarily mediated by the activation of caspase-3/-7, whereas canonical pyroptosis involves caspase-1/-4/-5/-11. Emerging evidence demonstrates that caspase-3 can alternatively trigger pyroptotic cancer cell death through GSDME cleavage, establishing a molecular bridge between apoptotic and pyroptotic pathways [34,35]. GSDME switches apoptotic cell death to pyroptosis, with its expression level determining the cell death modality in tumor cells. Upon high expression, activated caspase-3 cleaves GSDME to liberate its N-terminal domain, which perforates the plasma membrane, thereby inducing tumor pyroptosis [36]. The DNA methylation inhibitor DEC effectively reverses GSDME epigenetic silencing. Furthermore, DEC combined with chemotherapy (SN38) [37] or PDT enhances adaptive and innate immune responses in metastatic breast cancer models through apoptosis-pyroptosis transition, providing a strategic approach to overcome immunotherapy resistance. This study demonstrated that HPPH-N2 amplifies ROS-mediated caspase-3 activation and GSDME cleavage. This cascade generated GSDME-N terminal domains that formed plasma membrane pores, inducing pyroptotic cell death. This finding is consistent with the work by Ding et al., demonstrating that the ROS-Caspase-3-GSDME axis constitutes a critical pathway for inducing pyroptosis in tumor cells [38]. These results validate photo-controlled pyroptosis (Photo-Pyro) as a novel strategy for eliciting robust anti-tumor immunity [20].

Immunotherapy efficacy hinges on dendritic cell maturation, cytotoxic T lymphocyte infiltration, and immune memory establishment [39]. The synergistic interplay between PDT-induced ICD and pyroptosis offers a transformative strategy to potentiate breast cancer immunotherapy. PDT-generated ROS amplify ICD through endoplasmic reticulum stress-mediated DAMPs release and antigen cross-presentation, while GSH-depleting HPPH-N2 further enhance ROS accumulation, intensifying immunogenic signaling. Subsequently, PDT-induced apoptosis-to-pyroptosis transition releases pro-inflammatory cytokines including IL-1β and IL-18 [40]. These cytokines recruit cytotoxic T cells while remodeling immunosuppressive TNBC microenvironments. Pyroptosis further establishes chemokine gradients to overcome T-cell exclusion [41]. Ultimately, light-controlled pyroptosis induces ICD that enhances tumor antigen presentation for in situ vaccination [42], bridging innate cytotoxicity with adaptive immune activation. In murine models, combining this strategy with PD-1 blockade (which involves anti-PD-1 antibodies releasing T-cell suppression) suppressed 4T1 tumor recurrence through sustained immune memory formation.

The intrinsically complex biological features and elevated interstitial pressure of solid tumors impede effective delivery of nanomedicines [43].In situ therapy represents a promising alternative for optimizing cancer immunotherapy by enabling precise and sustained tumor treatment. While intratumoral injections improve drug bioavailability, their efficacy depends on operator skill and imaging guidance [44]. Advances in biomedical engineering, including self-illuminating in situ hydrogel with immune-adjuvant [45], polymer implants [46], and MN systems, offer precision alternatives [47]. Our MN platform delivered PLGA-SS-PLGA nanoparticles to epidermal/dermal layers rich in antigen-presenting cells, enabling sustained drug release via GSH-responsive and polymer degradation [48]. In addition, this approach maintained therapeutic drug concentrations while minimizing administration frequency, outperforming systemic venous delivery in both tumor retention and patient compliance. Emerging MN technologies continue to address photosensitizer delivery challenges. Recent innovations like the cryo-microneedle patch for HMME/catalase co-delivery exemplify progress in photosensitizer stabilization and tumor-targeted release [49].

Furthermore, inspired by microneedles loaded with sparfloxacin and ZnMnS nanoparticles, which significantly inhibit tumor growth, reduce pulmonary metastasis, and exhibit potent antibacterial/antibiofilm properties, we propose developing multifunctional MNs for combined antitumor therapy and postoperative infection prevention [50].In summary, our microneedle-based co-delivery platform for HPPH-ss-NPs and DEC demonstrates potent suppression of breast tumor growth, postoperative recurrence, and metastasis. This strategy shows broad potential applicability and may be translatable to melanoma, squamous cell carcinoma, and other superficial tumors. However, it is critical to consider that tumors in murine breast cancer models are readily generated yet often lack the genetic complexity and phenotypic heterogeneity of human breast cancers, with additional disconnects in tumor microenvironment representation [51].Future studies will necessitate multi-model validation using PDX or organoid platforms [52].Subsequent work will also prioritize optimization of microneedle materials to achieve enhanced tissue penetration depth and real-time release monitoring capabilities.

## 5 Conclusion

This study presents a light-controlled pyroptosis strategy that synergizes PDT-induced ICD with decitabine-mediated pyroptosis to overcome immunosuppression in breast cancer. By engineering redox-responsive PLGA-SS-PLGA nanoparticles, we simultaneously enhanced PDT efficacy through GSH depletion and triggered caspase-3-dependent pyroptosis via GSDME restoration. This dual mechanism remodeled the tumor microenvironment by coupling antigen release from ICD with pyroptosis-driven inflammatory activation, effectively recruiting cytotoxic T cells and potentiating checkpoint blockade therapy. The integration of microneedle-mediated co-delivery ensured localized therapeutic action and sustained immune activation, addressing stromal barriers and metastatic recurrence.

## CRediT authorship contribution statement

**Hang Yu:** Writing – original draft, Visualization, Validation, Supervision, Software, Resources, Methodology, Investigation, Funding acquisition, Data curation, Conceptualization. **Yiqing Chen:** Writing – original draft, Validation, Software, Methodology, Investigation, Funding acquisition, Data curation, Conceptualization. **Jinjin Yin:** Methodology, Investigation, Data curation, Conceptualization. **Zhongwen Yuan:** Supervision, Project administration, Funding acquisition. **Senling Feng:** Project administration, Methodology, Investigation, Conceptualization. **Yanrong Duan:** Software, Methodology, Investigation, Data curation. **Pengke Yan:** Supervision, Project administration, Funding acquisition, Conceptualization. **Shengyao Liu:** Project administration, Methodology, Investigation, Data curation, Conceptualization. **Wenting Zhu:** Writing – review & editing, Writing – original draft, Visualization, Validation, Supervision, Software, Resources, Project administration, Methodology, Investigation, Funding acquisition, Formal analysis, Data curation, Conceptualization.

## Ethics approval and consent to participate

All animal studies were performed in strict accordance with legal mandates and national guidelines for the care and maintenance of laboratory animals. The Ethics Committee of the Third Affiliated Hospital of Guangzhou Medical University approved all experimental protocols(2024-140).

## Consent for publication

All authors agree to be published.

## Funding

This work was supported by the 10.13039/501100003453Natural Science Foundation of Guangdong Province (No. 2025A1515011942), Plan on Enhancing Scientific Research in GMU (2024SRP104, 2025SRP023), Guangzhou Basic and Applied Basic Research Foundation (2024A03J0189, 2024A03J0188), and the Guangzhou Municipal Bureau of Science and Technology Key Research and Development Program (2024B03J0056), Tertiary Education Scientific research project of Guangzhou Municipal Education Bureau (2024312182), Medical Scientific Research Foundation of Guangdong Province (A2024212), Guangzhou Regional Major Science and Technology Project on Traditional Chinese Medicine (2025QN014, 2025CX014).

## Declaration of competing interest

The authors declare that they have no known competing financial interests or personal relationships that could have appeared to influence the work reported in this paper.

## Data Availability

Data will be made available on request.
