# Supplementary material for: Light-controlled pyroptosis via redox-responsive microneedles enhances photodynamic–epigenetic immunotherapy in breast cancer
Source: Mater Today Bio. 2025 Jul 31;34:102158. doi: 10.1016/j.mtbio.2025.102158 (PMC12536554; doi:10.1016/j.mtbio.2025.102158)
Supplement: Multimedia component 1 [file mmc1.docx]

# Supplementary Material

## **Light-Controlled Pyroptosis via Redox-Responsive Microneedles Enhances Photodynamic–Epigenetic Immunotherapy in Breast Cancer**


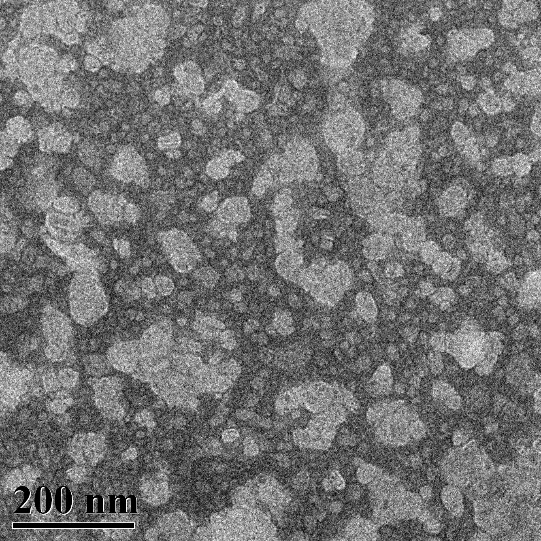


Fig. S 1 TEM images of N2 after 10mM GSH exposure.


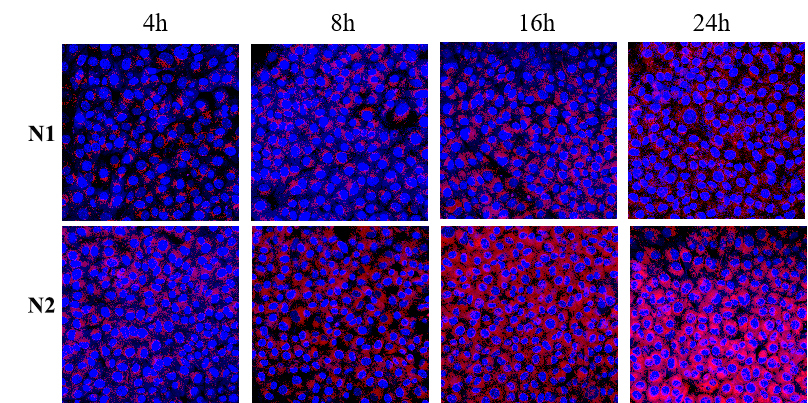


Fig. S 2 CLSM images of 4T1 cells’ uptake of different nanoparticle formulations


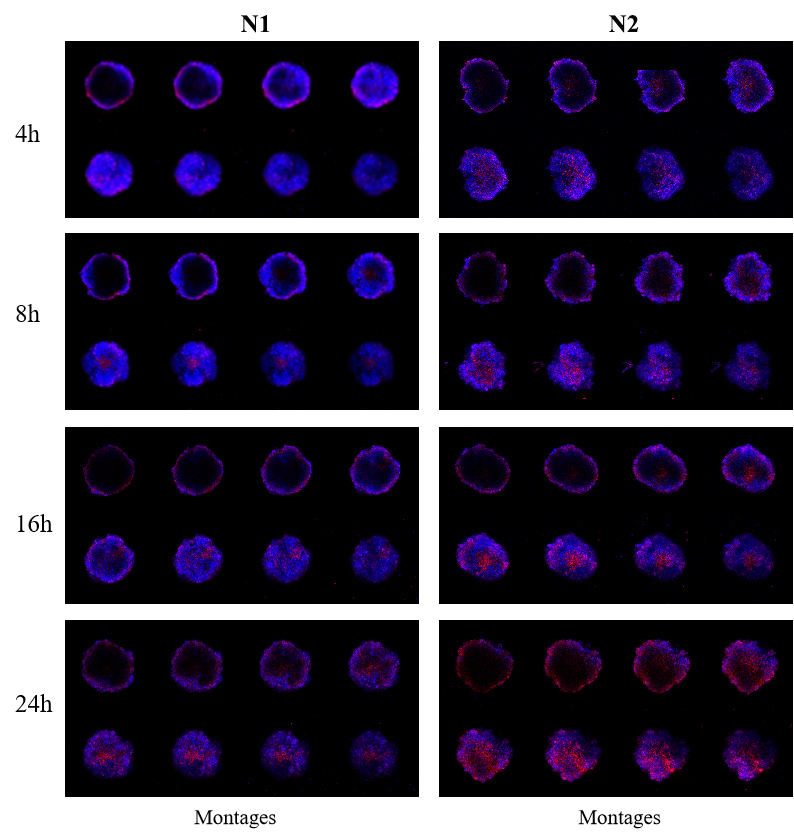


Fig. S 3 CLSM images of the uptake of different nanoparticle formulations by 3D spheroids of 4T1 cells.
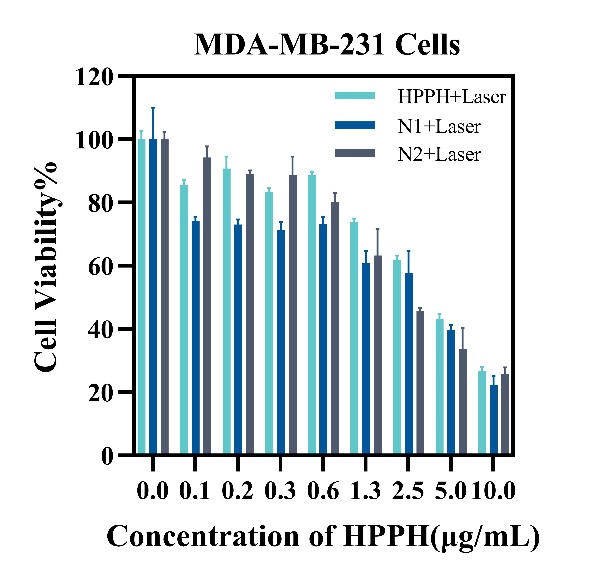


Fig. S 4 Cytotoxicity results for HPPH and its formulations against MDA-MB-231 cells.


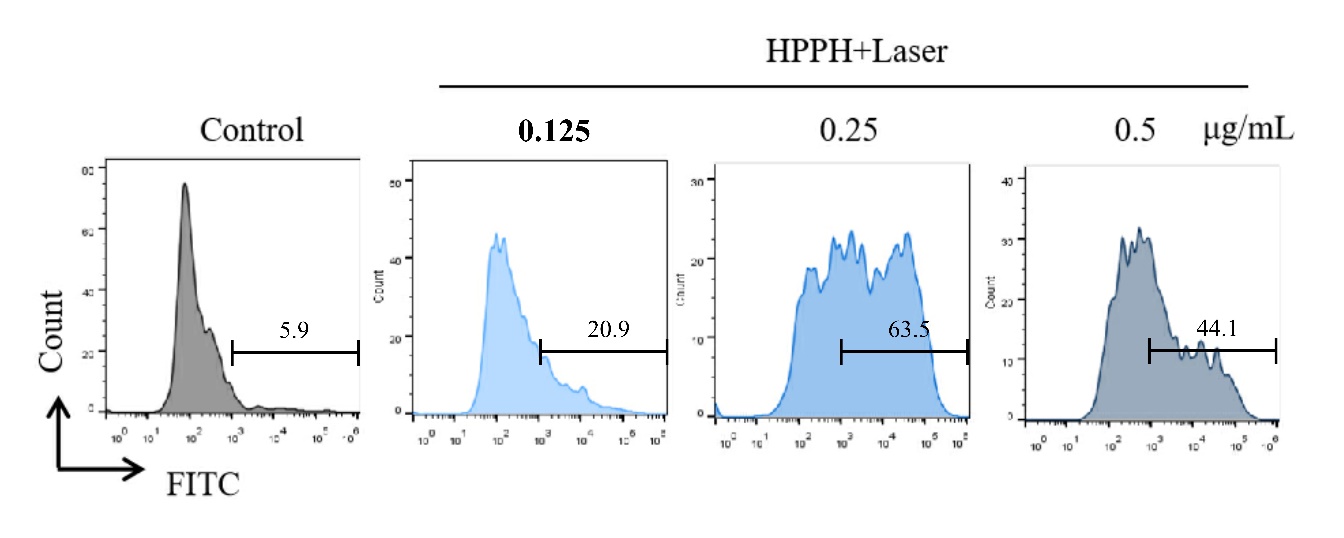


Fig. S 5 Flow cytometry analysis of ROS levels in 4T1 cells treated with different concentrations of HPPH.


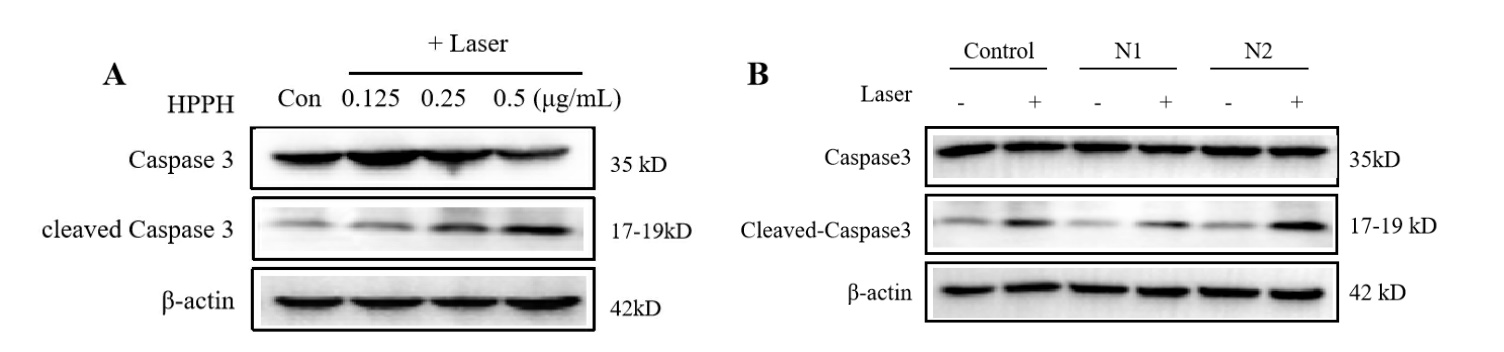


Fig. S 6 (A)Western blot images of Caspase3 and cleaved Caspase 3 expressed in 4T1 cells effected by different concentrations of HPPH . (B)Western blot images of Caspase3 and cleaved Caspase 3 in 4T1 cells treated with different nanoparticle formulations of HPPH.


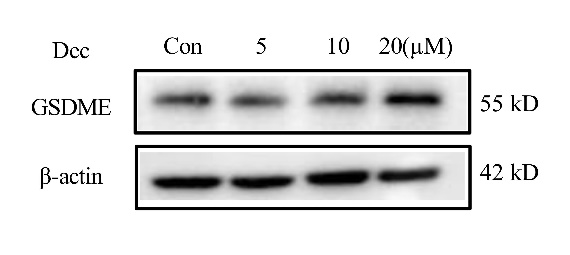


Fig. S 7 Western blot images of GSDME expressed in 4T1 cells treated with different concentrations of DEC.


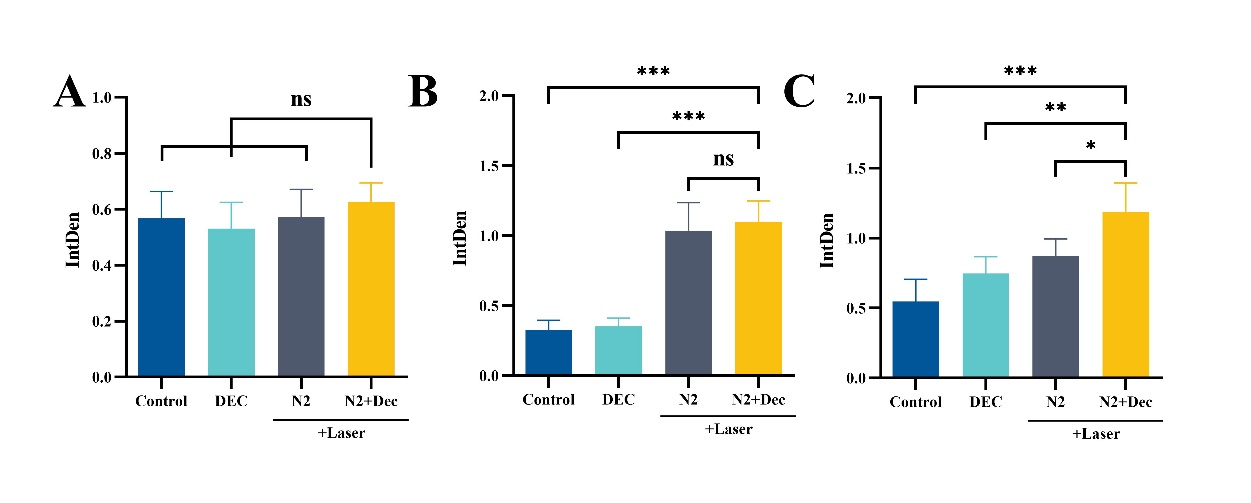


Fig. S 8 Statistical analysis for Western blot images of Caspase3 (A), cleaved Caspase 3 (B) and GSDME-N (A) in 4T1 cells treated with HPPH-ss-N2 combined with Dec.


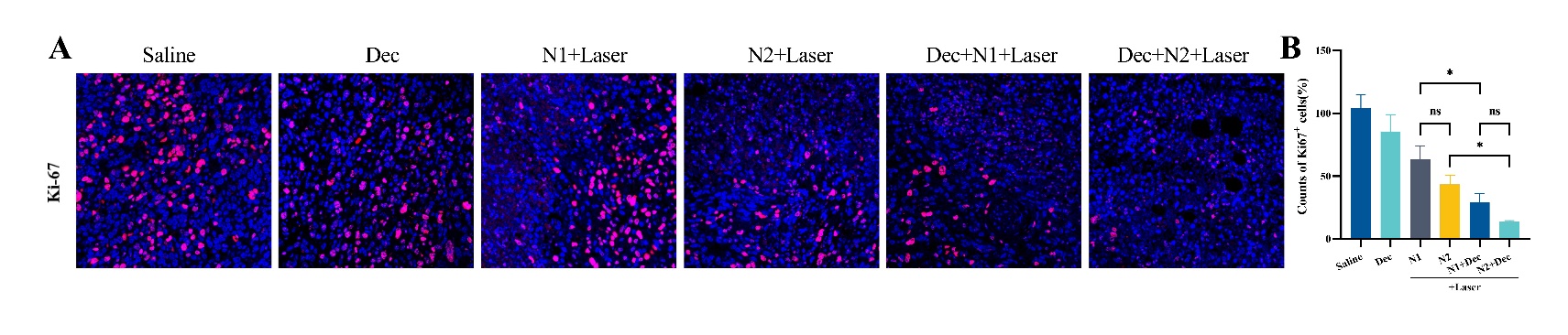


Fig. S 9 (A) CLSM images of Ki67 staining in tumor tissues and (B) their statistical analysis .


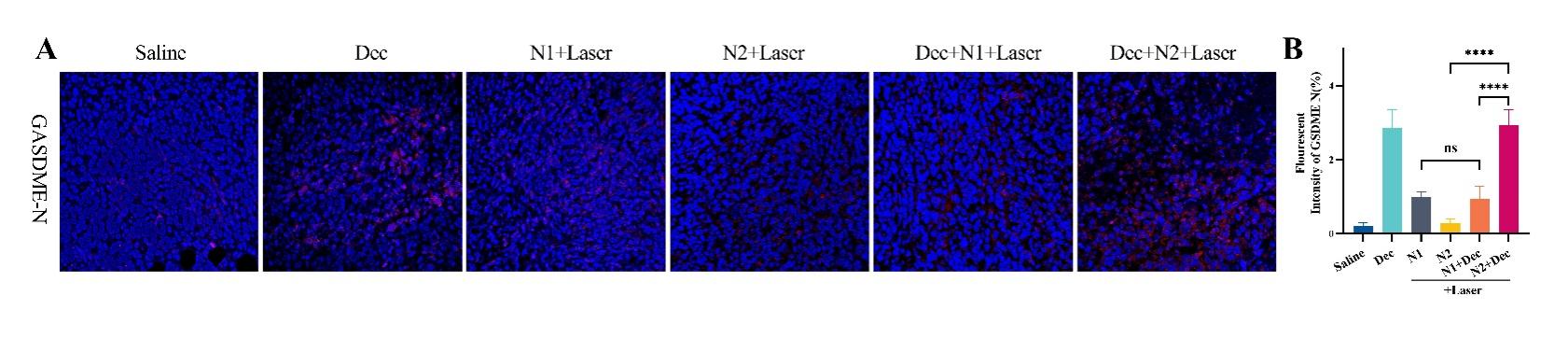


Fig. S 10 (A) Light photograph of the tumor sections stained by GSDME-N and (B) corresponding data analyze .


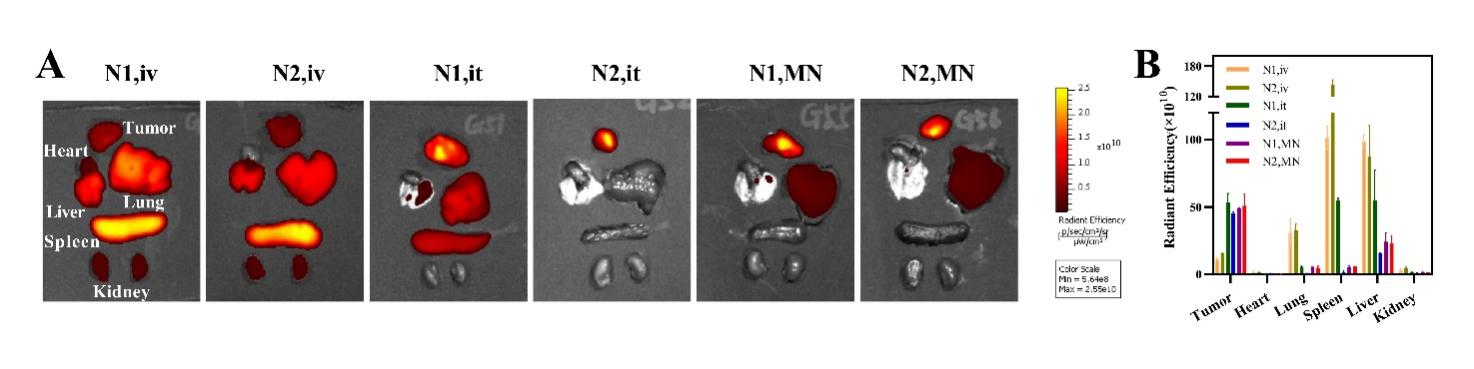


Fig. S 11 (A) Fluorescence distribution images of ex vivo organs and (B) its statistical analysis.


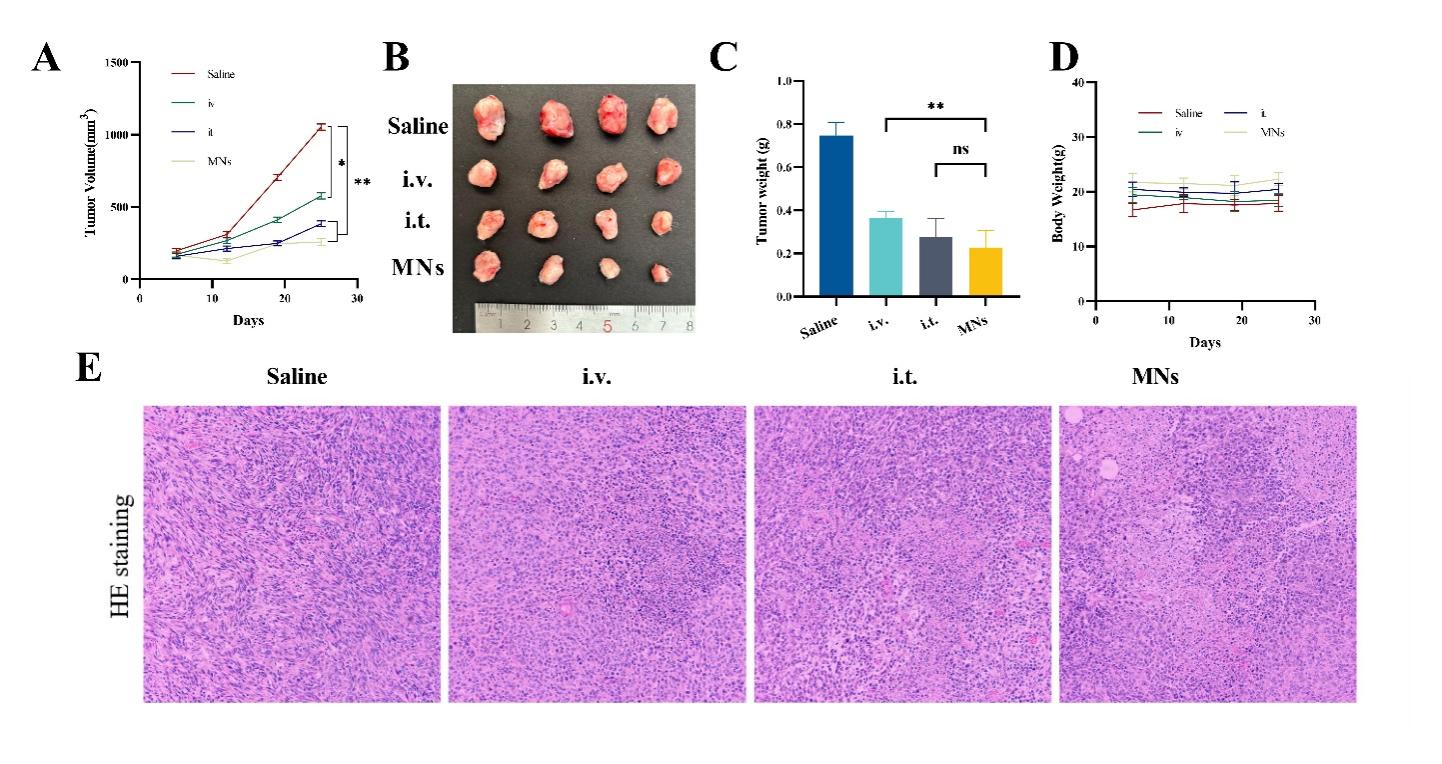


Fig. S 12 Therapeutic effects of different administration methods on a 4T1 breast cancer mouse model. (A)Changes in tumor volume. (B)Images of ex vivo tumors . (C)Weights of ex vivo tumors. (D) Body weight of mice. (E) H&E staining images of ex vivo tumor tissue sections .


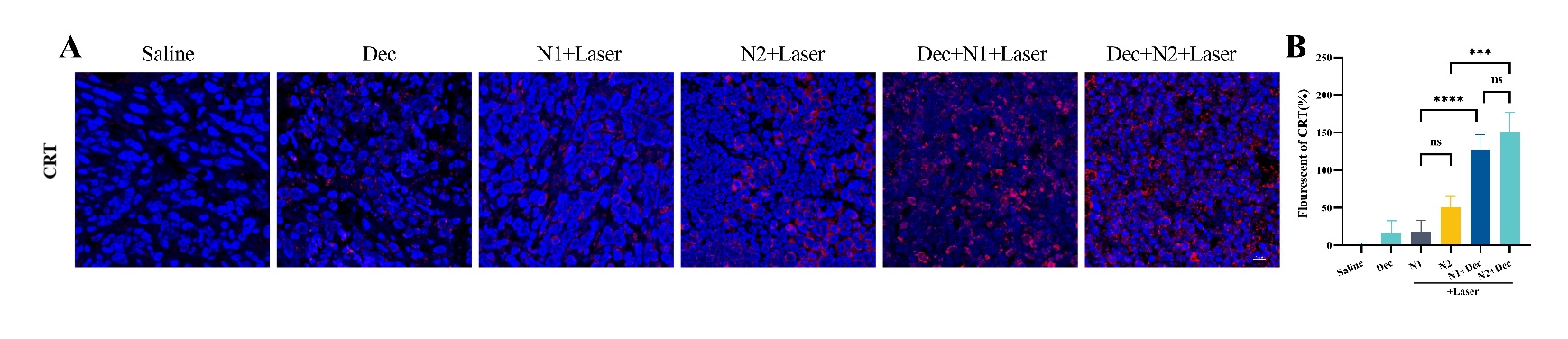


Fig. S 13 (A) CLSM images of CRT staining in tumor tissues and (B) their statistical analysis.


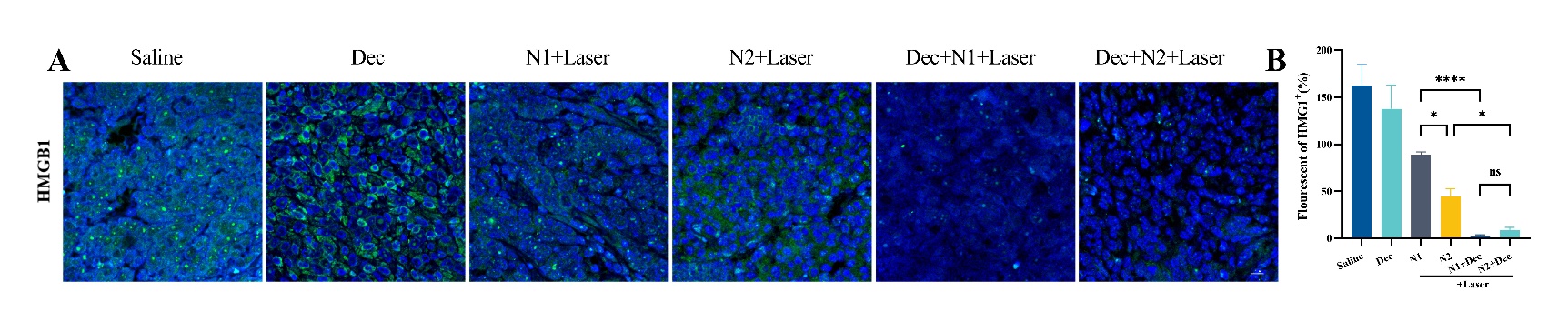


Fig. S 14 (A) CLSM images of HMGB1 staining in tumor tissues and (B) their statistical analysis .


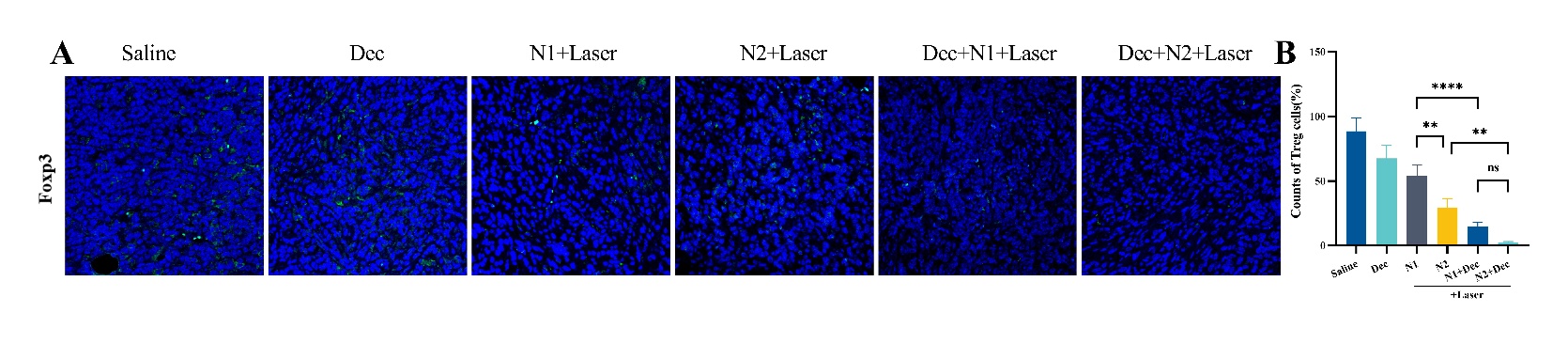


Fig. S 15 (A) CLSM images of Foxp3 staining in tumor tissues and (B) their statistical analysis .


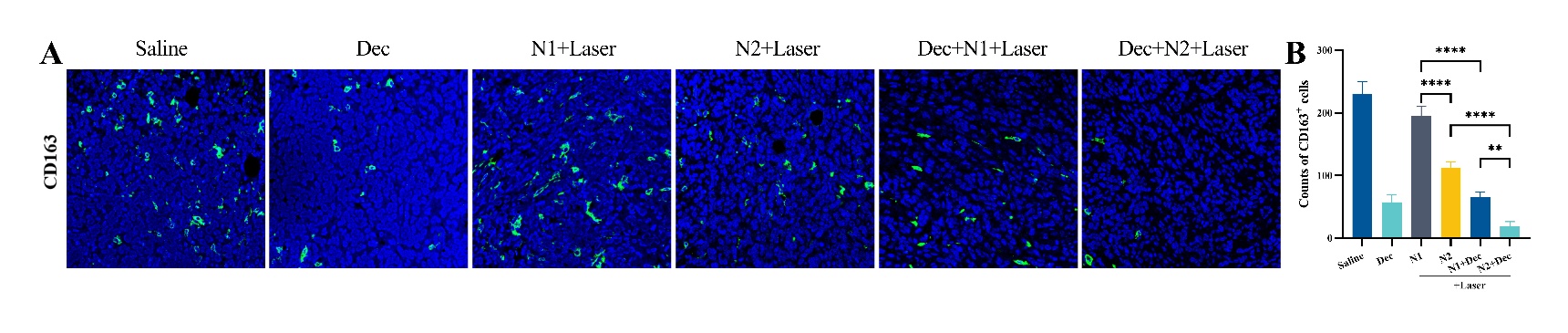


Fig. S 16 (A) CLSM images of CD163 staining in tumor tissues and (B) their statistical analysis .


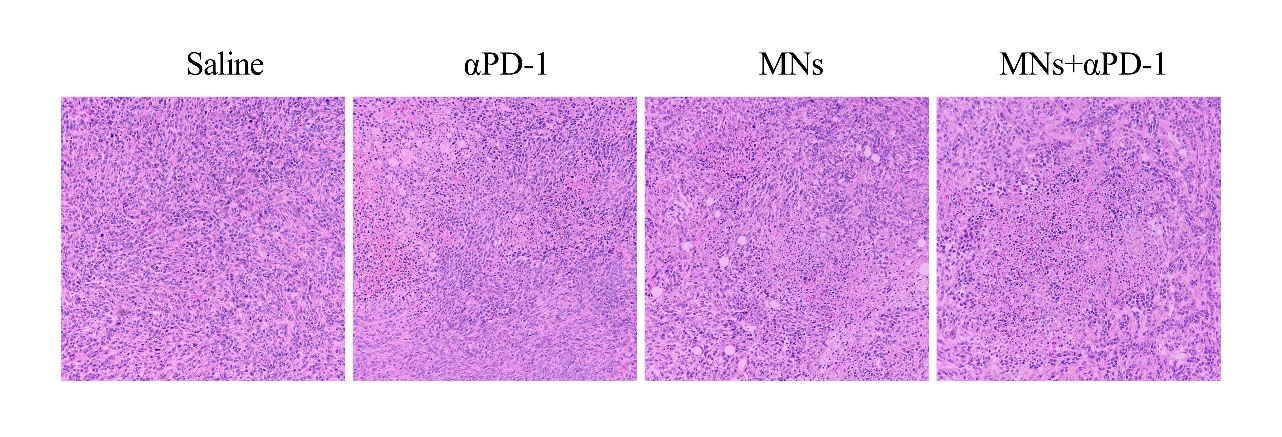


Fig. S 17 H&E staining images of recurrent tumor tissue sections.


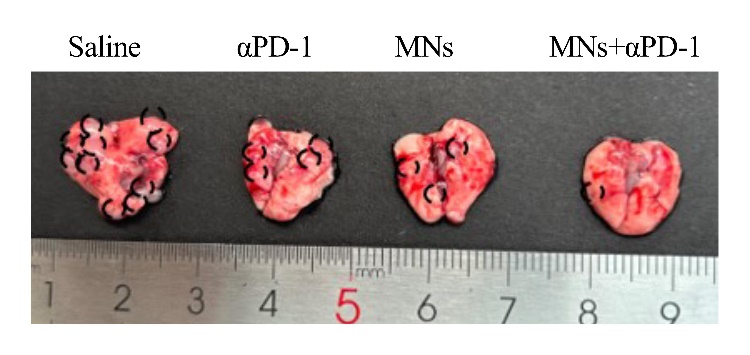


Fig. S 18 Images of the lungs from 4T1 breast cancer lung metastasis mouse model.


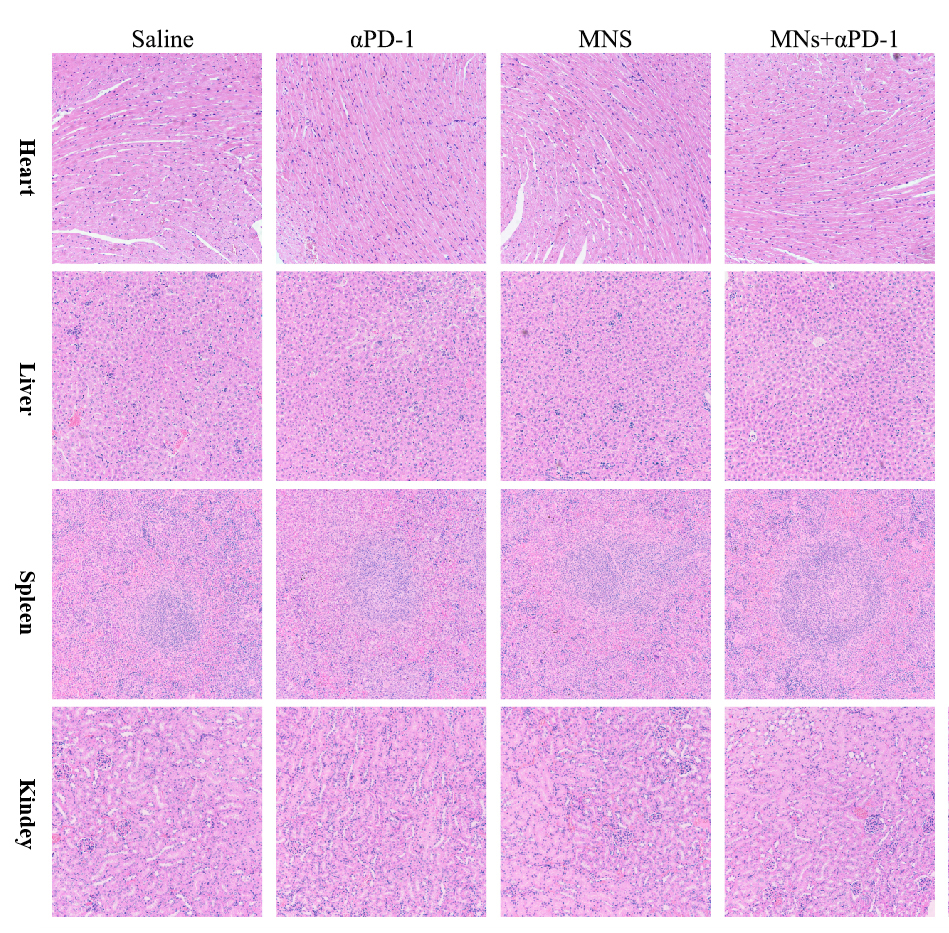


Fig. S 19 H&E staining images of main organs sections from a 4T1 tumor mouse model.


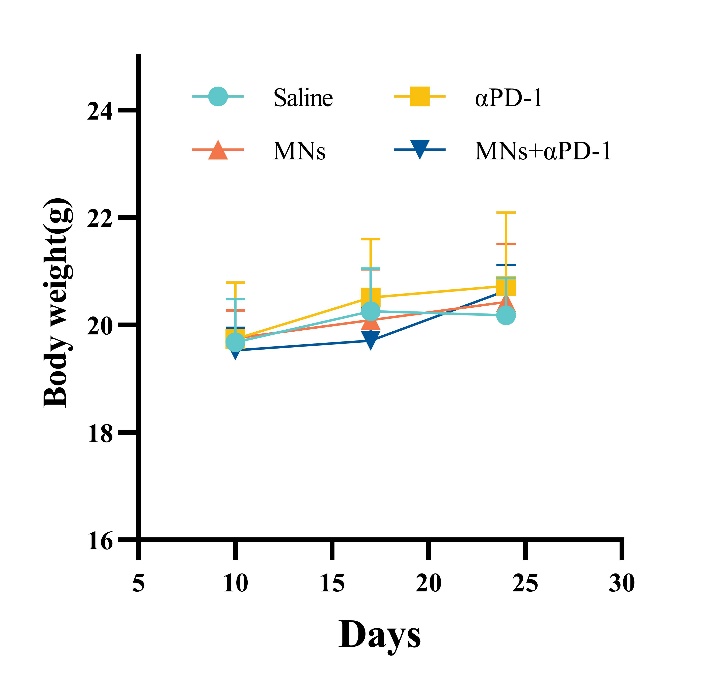


Fig. S 20 Body weight of recurrent tumor mice model .
